# Adjunctive esketamine in propofol-based sedation for gastrointestinal endoscopy: a systematic review and meta-analysis of randomized trials

**DOI:** 10.3389/fphar.2025.1662057

**Published:** 2025-11-25

**Authors:** Jiazheng Qi, Mengqiang Luo, Wenru Zong, Lingjing Zhang, Baoxuan Chen, Xiaoyu Yang, Bo Xu, Xu Zhao

**Affiliations:** 1 Department of Anesthesiology, Huashan Hospital, Fudan University, Shanghai, China; 2 Department of Anesthesiology, Second Affiliated Hospital of Zhejiang University School of Medicine, Zhejiang, China; 3 Department of Anesthesiology, The First Affiliated Hospital, Sun Yat-sen University, Guangzhou, China

**Keywords:** esketamine, gastrointestinal endoscopy, hemodynamic, propofol, adverse respiratory events, dizziness

## Abstract

**Background:**

While propofol is widely used for gastrointestinal endoscopic sedation, its cardiovascular and respiratory side effects and lack of analgesia can compromise safety and comfort. Esketamine provides both sedation and analgesia with minimal hemodynamic or respiratory impact. Combining esketamine with propofol may miti-gate propofol’s adverse effects while enhancing sedation quality. However, the com-bination’s overall safety and efficacy remain inconclusive.

**Methods:**

This systematic review and meta-analysis compared propofol-based sedation with *versus* without intravenous esketamine in gastrointestinal endoscopy, synthesizing evidence from randomized controlled trials. The primary outcome was the incidence of hypotension. Secondary outcomes included intraoperative adverse respiratory events, propofol consumption, involuntary movement, hypertension, arrhythmias, PONV recovery times, and dizziness.

**Results:**

Eighteen trials were included in the analysis. Additional esketamine significantly reduced the incidence of hypotension (risk ratio [RR]: 0.32; 95% confidence interval [CI]: 0.24 to 0.43; P < 0.01; I^2^ = 44.4%; moderate quality). The addition of esketamine to propofol can reduce the incidence of adverse respiratory events (RR: 0.57, 95% CI: 0.38 to 0.86; P < 0.01; I^2^ = 67.8%; moderate quality). Esketamine added to propofol decreased involuntary movement (RR: 0.61, 95% CI: 0.42 to 0.92; P = 0.02; I^2^ = 77.2%; low quality) and reduced the propofol consumption (mean difference [MD]: −0.94, 95% CI: −1.53 to −0.35 mg/kg; P < 0.01; I^2^ = 96.2%; low quality). No significant differences were found for hypertension, arrhythmias, PONV, recovery time or dizziness.

**Conclusion:**

Supplementing propofol-based sedation with esketamine reduced the risk of hypotension and adverse respiratory events, without increasing cardiovascular complications, or extending recovery-time.

**Systematic Review Registration:**

https://www.crd.york.ac.uk/PROSPERO/, identifier CRD420251030940.

## Introduction

In 2016, 9,808,182 gastroscopies and 4,350,950 colonoscopies were performed nationwide, highlighting the increasing prevalence of sedative procedures alongside improving living standards and health awareness (Zhou et al., 2021). However, the risks and mortality rates of procedural sedation in nontraditional operating rooms were not ignorable (Choi et al., 2018). It has become increasingly complex to evaluate the technological factors and level of sedation associated with adverse outcomes, especially in high-risk patients, e.g., elderly and pediatric patients and in those with comorbidities (Kim, 2020).

Over the past few decades, benzodiazepines, opioids, etomidate, and propofol have greatly advanced painless procedural sedation (Olkkola et al., 2008; Devlin and Roberts, 2009). Propofol is associated with few toxic side effects and a short awakening time (Bateman and Kesselheim, 2015), and therefore is the cornerstone sedative for gastrointestinal endoscopy. However, propofol causes dose-dependent cardiovascular and respiratory depression; its deep sedation is not directly reversible, which can lead to hemodynamic instability, hypoventilation, or in rare cases, the need for cardiopulmonary support during procedural sedation (Cote et al., 2010). In addition, propofol lacks intrinsic analgesia, often necessitating rescue dosing, and thereby reducing patient comfort (Bandschapp et al., 2010).

Considering the increasing demand for gastrointestinal endoscopy worldwide, safer and more efficient sedation regimens are being actively explored, with esketamine attracting growing attention in recent years. Esketamine is a right-handed optical isomer of ketamine, with the same basic properties as ketamine (Hirota and Lambert, 2022). However, esketamine has a higher affinity for μ-opioid receptors, a stronger analgesic effect, and induces fewer psychomimetic symptoms than ketamine (Zanos et al., 2018; Himmelseher and Pfenninger, 1998). Esketamine combines potent NMDA-mediated analgesia with dissociative sedation while exerting minimal cardiorespiratory suppression (Jonkman et al., 2018; Eberl et al., 2020). Making it an attractive adjunct in endoscopic procedures. Several randomized trials have evaluated intravenous esketamine within propofol-based protocols for gastrointestinal endoscopy, reporting benefits such as reduced propofol requirements and greater hemodynamic stability (Yang et al., 2022b; Zhan et al., 2022). However, the findings remain inconsistent, in part because study doses, co-medications, and outcome definitions differ widely (Chen et al., 2023a). Consequently, the overall safety and efficacy of esketamine–propofol sedation for gastrointestinal endoscopy are still uncertain and merit systematic appraisal.

In the current systematic review and meta-analysis, we aimed to evaluate the effect of intravenous esketamine on hemodynamics, adverse respiratory events, and postoperative recovery after procedural sedation based on randomized clinical trials (RCTs).

## Methods

This study was conducted and the data are reported in accordance with the Preferred Reporting Items for Systematic Reviews and Meta-Analyses (PRISMA) Statement. A predefined protocol was prospectively registered at the International Prospective Registry of Systematic Reviews (PROSPERO; CRD420251030940).

### Search strategy and selection criteria

We systematically searched for relevant studies indexed in the MEDLINE (PubMed), Web of Science, and Cochrane Central Register of Controlled Trials (CENTRAL) databases from their respective inception dates through March 30, 2025. Additionally, we updated our search results on August 20, 2025, and conducted searches in three Chinese databases (CNKI, VIP, and Wanfang) to provide a more comprehensive estimate. In addition, we searched the reference lists of review articles and included additional trials that were not initially identified in our electronic search of the primary databases. To strengthen the comprehensiveness and scope of application of the included studies, no limitations were imposed on the age of the individuals included in the analysis, and no language restrictions were applied. The following search terms were used: esketamine, S-ketamine; sedation; sedative surgery; painless; gastrointestinal endoscopy, digestive system endoscopy and related Mesh terms. The complete search strategy is summarized in Supplementary Appendix S1.

We included trials that compared intravenous esketamine combined with propofol to propofol-based sedation during gastrointestinal endoscopy. No restrictions were imposed on the age of patients. The intervention could be using the combination of intravenous esketamine added to propofol-based sedation. The comparator was propofol or its primary components. Two authors independently screened the titles and abstracts for eligible full-text articles. Any disagreements regarding the inclusion of a trial were resolved by consulting a third author. Two authors independently collected data using a standard data collection template, including information about the authors, publication date, type of surgery, intervention group, control group, mode of anesthesia, dosage of each anesthetic used, and outcome data.

### Measurement of outcome data

The primary outcome was the incidence of intraoperative hypotension. Secondary outcomes included adverse respiratory events, involuntary movement, propofol consumption, other intraoperative hemodynamic index (hypertension and arrhythmia), PONV, postoperative recovery time, and the frequency of dizziness after awakening. Hypotension was defined as a decrease in mean arterial pressure from baseline of more than 20% and systolic blood pressure below 80 mmHg or 80% of the baseline value, as previously described (Zhan et al., 2022; Chen et al., 2022a; Feng et al., 2022; Wang et al., 2022; Eberl et al., 2020; Lu et al., 2023). Adverse respiratory events, which was defined as hypoxemia (oxygen saturation level below 95% based on pulse oximetry (Zhan et al., 2022; Chen et al., 2023a; Zhong et al., 2023; Zheng et al., 2023a)) lasting longer than 10s, laryngospasm, increased oxygen flow, asphyxia, and the requirement of mechanical ventilation during surgery (Yang et al., 2022a; Nie et al., 2023; Zheng et al., 2023a). Involuntary movement refers to unconscious muscle contractions or limb movements triggered by physiological reflexes (such as pharyngeal reflex or intestinal spasm) or painful stimuli during the examination (Liu et al., 2023). Propofol consumption was defined as the amount of propofol consumed per kilogram of body weight throughout the sedation process (Wang et al., 2022; Zheng et al., 2022). Hypertension was defined as blood pressure 30% higher than baseline values (Zhan et al., 2022). Arrhythmias included bradycardia, tachycardia, or other types of irregular heartbeat (Wang et al., 2022; Eberl et al., 2020). Postoperative recovery time was defined as the length of time required for a full restoration of consciousness after sedation/anesthesia, an Observer Assessment of Alertness/Sedation (OAA/S) score greater than 5, or a modified OAA/S score greater than 4 (Yang et al., 2022a; Eberl et al., 2020; Hirota and Lambert, 2022).

### Assessment of risk of bias (RoB)

Using the Cochrane Collaboration tool for randomized trials (available from http://handbook-5-1.cochrane.org/), two reviewers independently assessed the RoB due to the randomization process, deviation from the expected intervention, lack of outcome data, and risks related to the outcome measures and selection of reported outcomes. Any discrepancies were resolved through discussion or, if necessary, by involving a third author.

### Data synthesis and analysis

To ensure a high-quality meta-analysis was performed, data from each of the included studies were extracted by two different people and initially integrated into data tables by others in the group. Authors entered the data into Review Manager 5.3 software (version 5.3; The Nordic Cochrane Center, The Cochrane Collaboration, Copenhagen, Denmark) and R statistical software (version 4.2.1) following multiple rounds of proofreading. The means and standard deviations were entered for continuous variables, and the number of occurrences was entered for dichotomous variables. We investigated the statistical heterogeneity using the I^2^ statistic, defined as the percentage of total variability between studies resulting from heterogeneity rather than random chance. We used the guidelines from the Cochrane Handbook to quantify the degree of heterogeneity (Cumpston et al., 2019). Considering the variation and heterogeneity between different studies, we used the DerSimonian and Laird random-effects model for all statistical analyses (Chen et al., 2025). The quality of evidence for each pre-specified critical outcome was assessed using the GRADE approach, and the final evaluations were presented in Summary of Findings tables created with GRADEpro GDT.

The mean difference, 95% confidence interval (CI), and p-value were recorded for each continuous variable, whereas the RR, 95% CI, and p-value were recorded for each categorical variable. The cut-off for statistical significance was set at p < 0.05. If a sufficient number of publications was available (n ≥ 10), publication bias was assessed *via* funnel plots (visually) and more formally with the Egger’s test (Egger et al., 1997). In the presence of high heterogeneity (I^2^>50%), “leave-one-out” sensitivity analyses and additional analyses excluding high-risk articles were conducted for the primary outcome.

### Subgroup analysis and meta-regression analysis

Based on clinical considerations, subgroup analyses were performed to evaluate the outcomes of hypotension and adverse respiratory events according to: (1) inclusion of ASA class III patients, (2) concurrent use of opioids, (3) age exceeding 18 years, (4) surgical location and (5) the esketamine dosage. To further investigate the potential impact of methodological characteristics, meta-regression analyses were conducted to examine the associations between outcome measures and two continuous covariates: mean age of participants and sample size in each study.

## Results

**FIGURE 1:**
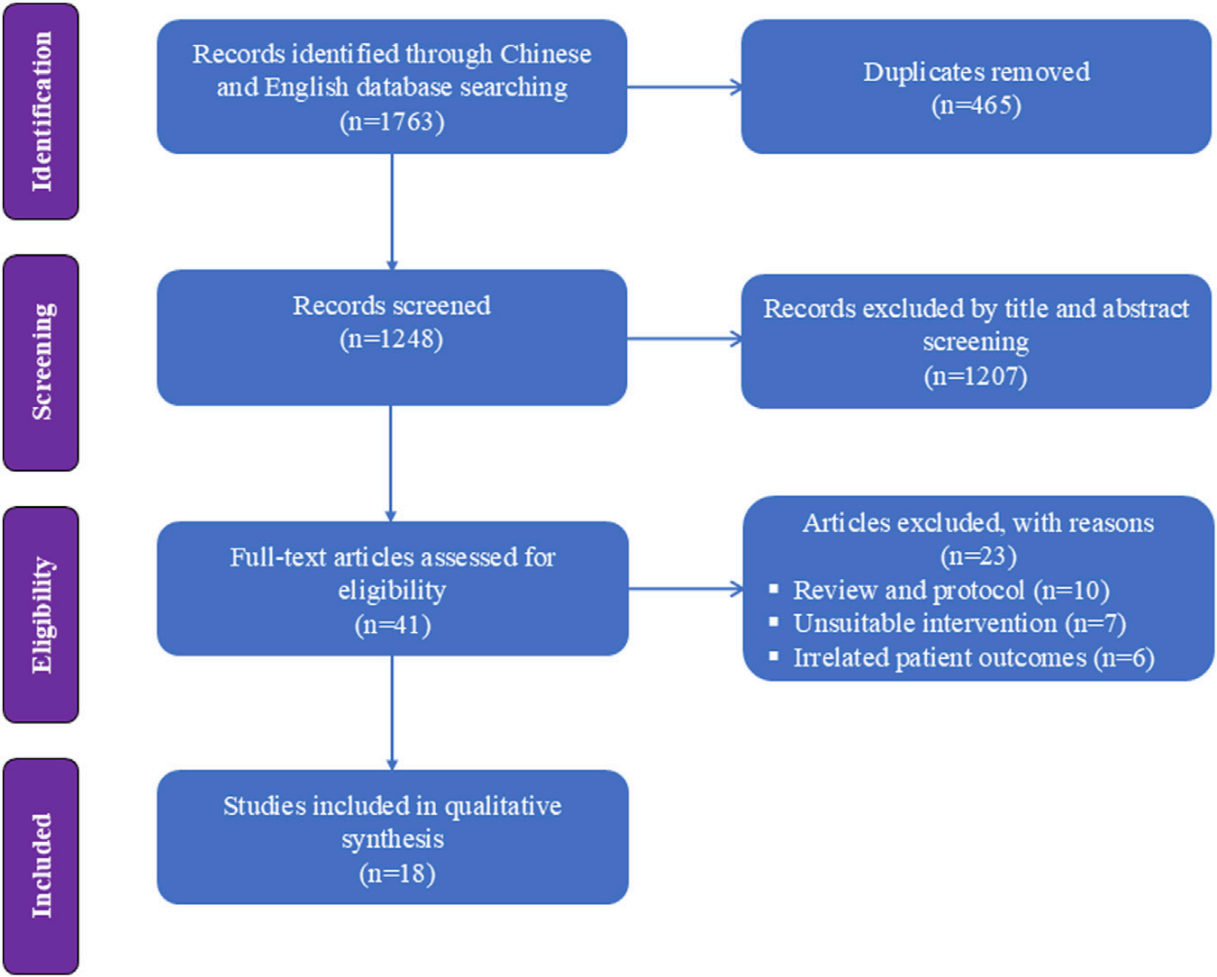
Flow diagram of the study selection process.

Figure 1 presents a flowchart regarding studies selected and excluded from the analysis. A total of 1763 relevant items were identified; after removing duplicates, the list was narrowed down to 1,248 relevant articles. Forty-one RCTs were assessed for full-text articles after further screening. After excluding another 10 studies for literature review (Barrett et al., 2020; Kamp et al., 2020; Khorassani and Talreja, 2020; Li et al., 2022; Lian et al., 2023; Mawere-Mubvumbi, 2023; Mihaljevic et al., 2020; Schep et al., 2023; Valencia-Arango et al., 2020; Yang et al., 2022a), 7 for unsuitable intervention (Annborn et al., 2023; Brinck et al., 2021; Dijkstra et al., 2022; Han et al., 2022; Huang et al., 2023a; Jiang et al., 2023; Hirota and Lambert, 2022), and 6 for unsuitable outcome (Liu et al., 2022; Min et al., 2023; Su et al., 2023; Tan et al., 2022; Xin et al., 2022; Yu et al., 2022), 18 RCTs were included in the analysis (Yang et al., 2022a; Zhan et al., 2022; Chen et al., 2023a; Feng et al., 2022; Wang et al., 2022; Liu et al., 2023; Zheng et al., 2022; Eberl et al., 2020; Song et al., 2022; Xu et al., 2022; Zheng et al., 2023a; Lu et al., 2023; Chen et al., 2022b; Ye et al., 2023; Liu et al., 2025; Xiao et al., 2024; Xue et al., 2025; Li et al., 2025). Eight of these articles (based on data from a total of 1,656 patients) involved painless gastrointestinal surgery (Yang et al., 2022a; Zhan et al., 2022; Feng et al., 2022; Zheng et al., 2022; Song et al., 2022; Zheng et al., 2023a; Lu et al., 2023; Chen et al., 2022b); Five article (based on 542 patients) involved gastroscopy (Wang et al., 2022; Liu et al., 2023; Xu et al., 2022; Xue et al., 2025; Li et al., 2025); Three studies (including 470 patients) reported the application of esketamine in colonoscopy (Ye et al., 2023; Liu et al., 2025; Xiao et al., 2024); one studies (including 162 patients) involved esketamine combined with propofol for endoscopic retrograde cholangiopancreatography (Eberl et al., 2020); one study (involving 100 patients) investigated painless endoscopic variceal ligation (Chen et al., 2023a). Detailed characteristics of the 18 included studies are shown in Table 1.

**TABLE 1:** Characteristics of the included studies.

Study	Mean age	Year	N	Type	Treatment group	Controlled group	Hemodynamic outcomes	Respiratory adverse event	Other outcomes
Chen et al. (2023a)	54.10	2023	100	EVL	(a) 0.2 mg/kg esketamine +1.5 mg/kg propofol (b) 0.3 mg/kg esketamine +1.5 mg/kg propofol (c) 0.4 mg/kg esketamine +1.5 mg/kg propofol, if needed, added 0.5 mg/kg propofol	1.5 mg/kg propofol +0.1 μg/kg sufentanil, if needed, added 0.5 mg/kg propofol	Hypotension, hypertension, arrhythmia	Hypoxemia	Propofol consumption, Recovery time
Chen et al. (2022b)	52.12	2022	82	GI	0.5 mg/kg esketamine +2 mg/kg propofol, if needed, added 1 mg/kg propofol	2 mg/kg propofol, if needed, added 1 mg/kg propofol	Arrhythmia	Respiratory depression	PONV, Recovery time
Eberl et al. (2020)	56.23	2020	162	ERCP	0.15 mg/kg esketamine +5 μg/mL propofol; when needed, add 0.5 μg/mL propofol and 0.05 mg/kg esketamine	2.0 μg/kg alfentanil +5 μg/mL propofol; when needed, add 0.5 μg/mL propofol and 1 μg/kg alfentanil	Hypotension, hypertension, arrhythmia	Hypoxemia	Propofol consumption, Recovery time
Feng et al. (2022)	52.88	2022	100	GI	(a) 0.15 mg/kg + esketamine, 2.5 μg/mL propofol (b) 0.25 mg/kg esketamine +2 ug/mL propofol (c) 0.5 mg/kg esketamine +1.5 μg/kg propofol, if needed, added/decrease 0.5–1 mg/kg propofol	3 μg/mL propofol, if needed, added/decrease 0.5–1 mg/kg propofol	Hypotension	Respiratory depression	Propofol consumption, Recovery time, PONV, dizziness Recovery time,
Liu et al. (2025)	61.35	2025	252	CS	0.2 mg/kg esketamine +1 mg/kg propofol, if needed, added 0.5 mg/kg propofol	1 mg/kg propofol, if needed, added 0.5 mg/kg propofol	Hypotension, arrhythmia	Hypoxemia	PONV, Propofol consumption,
Lu et al. (2023)	51.20	2023	172	GI	0.2 mg/kg esketamine +1.5–2 mg/kg propofol, if needed, added 0.5 mg/kg propofol	1.5–2 mg/kg propofol, if needed, added 0.5 mg/kg propofol	Hypotension, arrhythmia	Respiratory depression	PONV, Involuntary movement, Recovery time, dizziness
Liu et al. (2023)	47.36	2023	76	GS	0.2 mg/kg esketamine + 2 mg/kg propofol, if impossible, added 0.5 mg/kg propofol	2 mg/kg propofol, if impossible, added 0.5 mg/kg propofol	Hypotension, hypertension, arrhythmia	Hypoxemia	Propofol consumption, Involuntary movement, Recovery time, dizziness
Song et al. (2022)	43.84	2023	660	GI	0.15 mg/kg esketamine +0.5 mg/kg propofol +0.1 μg/kg sufentanil; if impossible, added 0.2–0.3 mg/kg propofol	0.5 mg/kg propofol +0.1 μg/kg sufentanil; if impossible, added 0.2–0.3 mg/kg propofol	Hypotension	Hypoxemia	PONV, dizziness
Wang et al. (2022)	9.42	2022	119	GS	(a) 0.3 mg/kg esketamine +2 mg/kg propofol (b) 0.5 mg/kg esketamine + 2 mg/kg propofol (c) 0.7 mg/kg esketamine +2 mg/kg propofol, if needed, added 1 mg/kg propofol	2 mg/kg propofol, if needed, added 1 mg/kg propofol	Hypotension	Hypoxemia	Propofol consumption, PONV, Recovery time, dizziness
Xiao et al. (2024)	48.30	2024	130	CS	0.2 mg/kg esketamine +0.5–0.8 mg/kg propofol, if needed, added 0.3 mg/kg propofol	2 mg/kg propofol, if needed, added 0.3 mg/kg propofol	Hypotension, Hypertension, arrhythmia	Hypoxemia,	PONV, Dizziness,
Xu et al. (2022)	40.62	2022	83	GS	0.3 mg/kg esketamine + propofol, if needed, added 20–50 mg propofol	0.05 mg/kg dezocine + propofol, if needed, added 20–50 mg propofol	Hypotension, arrhythmia	Hypoxemia	Propofol consumption, PONV, Recovery time
Xue et al. (2025)	44.06	2024	160	GS	0.2 mg/kg esketamine +2.0 mg/kg propofol, if needed, added 0.5 mg/kg propofol	2.0 mg/kg propofol, if needed, added 0.5 mg/kg propofol	Hypotension,	Hypoxemia, Respiratory depression	Involuntary movement, PONV, Dizziness,
Yang et al. (2022a)	68.83	2022	90	GI	(a) 0.25 mg/kg esketamine +2.5 μg/mL propofol (b) 0.5 mg/kg esketamine +2.5 μg/mL propofol	Normal saline +2.5 μg/mL propofol	Hypotension, arrhythmia	Respiratory depression	PONV
Ye et al. (2023)	50.77	2023	88	CS	0.2 mg/kg esketamine + 1–2 mg/kg propofol	1-2 mg/kg propofol + 0.1 µg/kg sufentanil	Hypotension, arrhythmia	Respiratory depression	Propofol consumption, PONV, Recovery time, dizziness
Zhan et al. (2022)	44.49	2022	260	GI	(a) 0.05 mg/kg esketamine +1.5 mg/kg propofol (b) 0.1 mg/kg esketamine +1.5 mg/kg propofol (c) 0.2 mg/kg esketamine +1.5 mg/kg propofol	Normal saline +1.5 mg/kg propofol	Hypotension, hypertension	Hypoxemia	Involuntary movement, PONV, Recovery time, dizziness
Li et al. (2025)	41.65	2023	104	GS	0.25 mg/kg esketamine +2 mg/kg propofol	Normal saline +2 mg/kg propofol	Hypotension; arrhythmia,	Hypoxemia	Involuntary movement, Recovery time
Zheng et al. (2022)	9.68	2022	92	GI	(a)0.25 mg/kg esketamine +2.5 mg/kg propofol, if impossible, add/decrease 0.75 mg/kg propofol (b)0.5 mg/kg esketamine +1.5 mg/kg propofol, if impossible, add/decrease 0.5 mg/kg propofol (c)1 mg/kg esketamine +1.5 mg/kg propofol, if impossible, add/decrease 0.25 mg/kg propofol	4.5 mg/kg propofol, if impossible, add/decrease 0.5 mg/kg propofol	Hypotension	Respiratory depression	Propofol consumption, PONV, dizziness
Zheng et al. (2023a)	8.30	2023	200	GI	0.5 mg/kg esketamine + 2 mg/kg propofol	0.2 mg/kg nalbuphine +2 mg/kg propofol	—	Respiratory depression	PONV, dizziness

GI, Gastrointestinal endoscopy; GS, Gastroscopy; ERCP, Endoscopic retrograde cholangiopancreatography; EVL, Endoscopic variceal ligation; CS, Colonoscopy.

**FIGURE 2:**
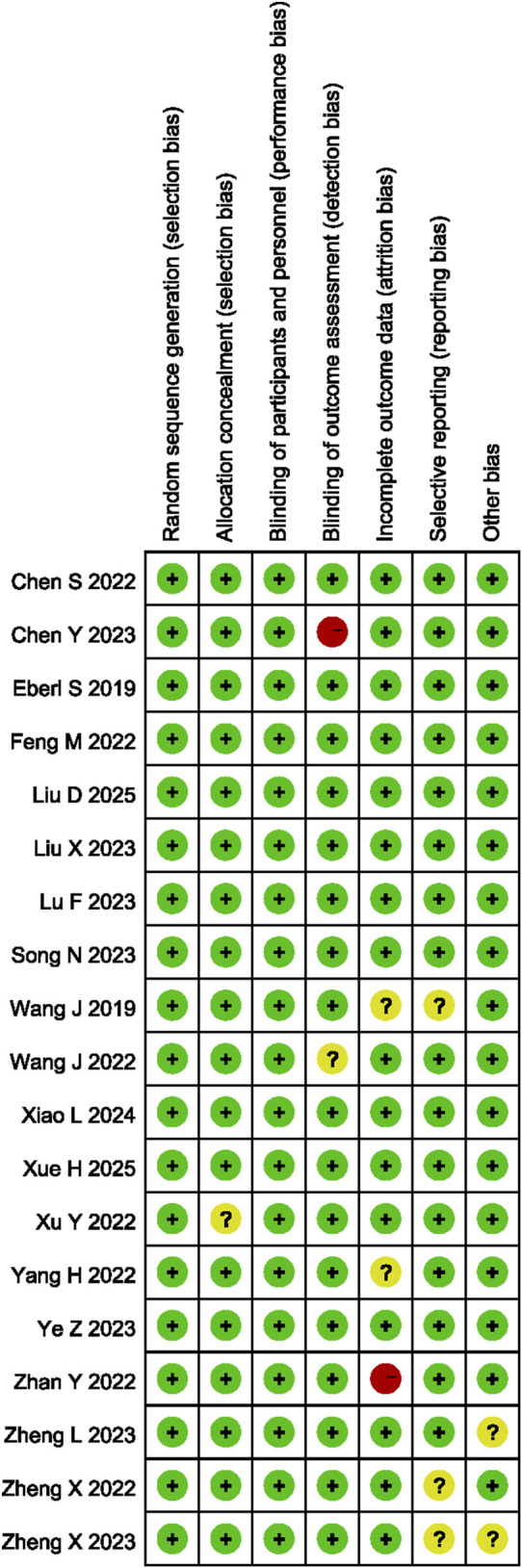
Risk of bias summary.

The RoB was assessed, as shown in Figure 2. Of the 18 studies selected, 16 exhibited low-level bias (Yang et al., 2022a; Feng et al., 2022; Wang et al., 2022; Liu et al., 2023; Zheng et al., 2022; Eberl et al., 2020; Song et al., 2022; Xu et al., 2022; Zheng et al., 2023a; Lu et al., 2023; Chen et al., 2022b; Ye et al., 2023; Liu et al., 2025; Xiao et al., 2024; Xue et al., 2025; Li et al., 2025). One of the 18 articles contained vague descriptions of the blinding procedure, as it lacked a description of how blinding was applied to the assessment of outcome measures (Chen et al., 2023a), another one lacked clarity regarding the results, as some of the outcome measures defined at the outset of the study were not reported (Zhan et al., 2022), which affected the quality of reporting and degraded the quality of the literature.

### Results related to the primary outcomes

#### Intraoperative hypotension during sedation

**FIGURE 3:**
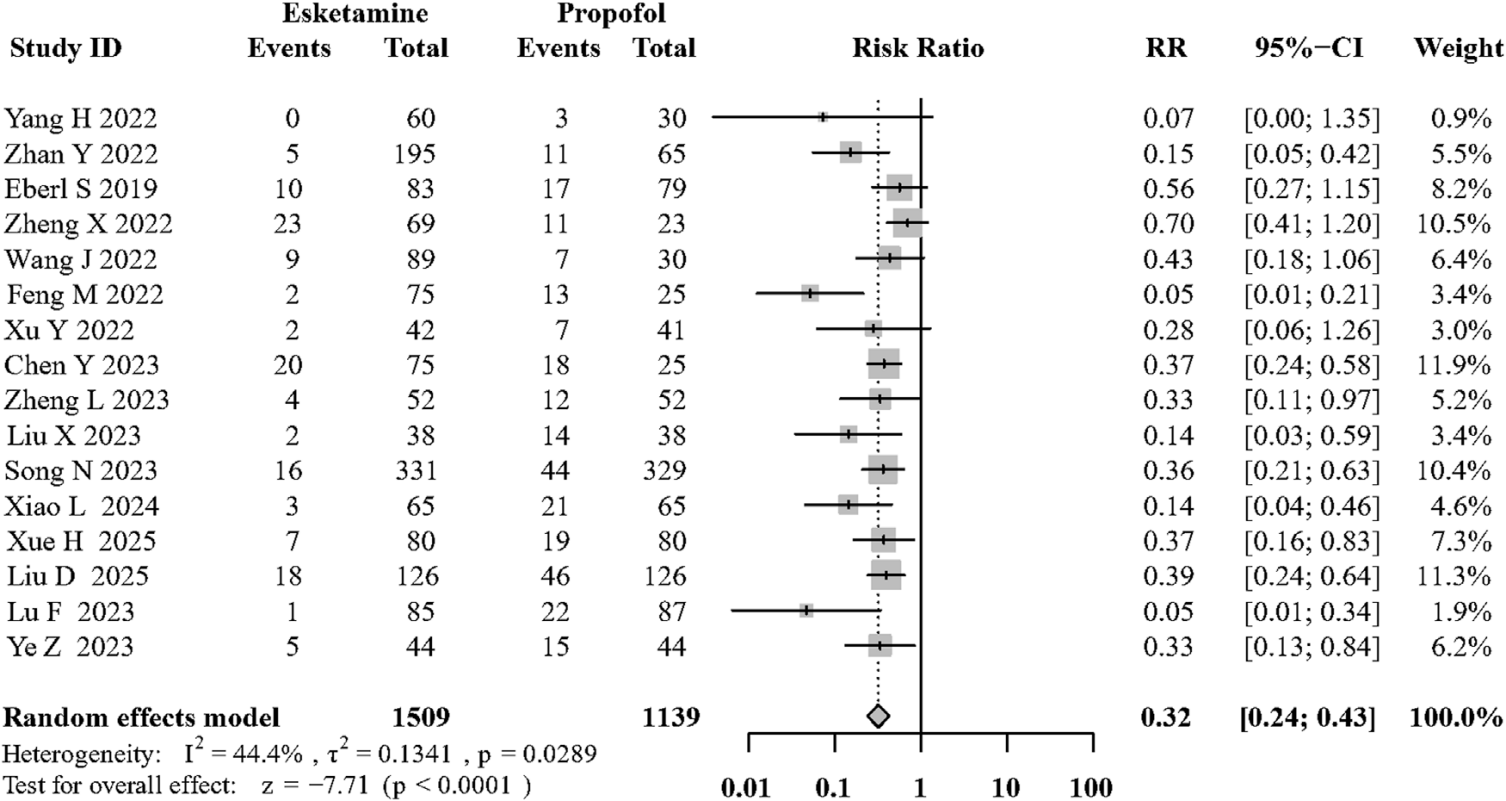
Forest plot of the incidence of intraoperative hypotension during sedation.

Sixteen of the included RCTs (Yang et al., 2022a; Zhan et al., 2022; Chen et al., 2023a; Feng et al., 2022; Wang et al., 2022; Liu et al., 2023; Zheng et al., 2022; Eberl et al., 2020; Song et al., 2022; Xu et al., 2022; Lu et al., 2023; Ye et al., 2023; Liu et al., 2025; Xiao et al., 2024; Xue et al., 2025; Li et al., 2025) evaluated its effects on hypotension during sedation. Compared with the propofol, the combination of intravenous esketamine and propofol significantly decreased the occurrence of intraoperative hypotension (risk ratio [RR]: 0.32; 95% CI:0.24 to 0.43, p < 0.01, I^2^ = 44.4%, moderate quality; Figure 3; Supplementary Table S1). Visual inspection of the funnel plot for the primary outcome revealed asymmetry, which was confirmed by Egger’s test (p = 0.002, Supplementary Figure S1A). To address potential publication bias, we applied the trim-and-fill method, which imputed 6 potentially missing studies. The adjusted pooled effect estimate remained statistically significant (RR = 0.41, 95% CI: 0.30–0.58), supporting the robustness of our primary findings (Supplementary Figures S3,S4). Furthermore, to more precisely capture the pharmacological effect of esketamine, we specifically reported mean arterial pressure (MAP) after induction. The addition of esketamine significantly increased post-induction MAP (MD: 8.24 mmHg; 95% CI: 5.36–11.12 (mmhg); p < 0.01; I^2^ = 64.9%; Supplementary Figure S18).

We conducted sensitivity analyses of the primary outcomes by excluding one study each time. Exclusion of any of the eleven articles did not cause a change in the overall results. The results of the “leave-one-out” sensitivity analyses are presented in Supplementary Figure S2. In addition, after excluding articles with high RoB, compared with the control group, the use of intravenous esketamine did decrease the occurrence of hypotension (RR: 0.32; 95% CI: 0.23 to 0.45, p < 0.01, I^2^ = 46.1%; Supplementary Figure S5).

### Results related to the secondary outcomes

#### Adverse respiratory events during sedation

**FIGURE 4:**
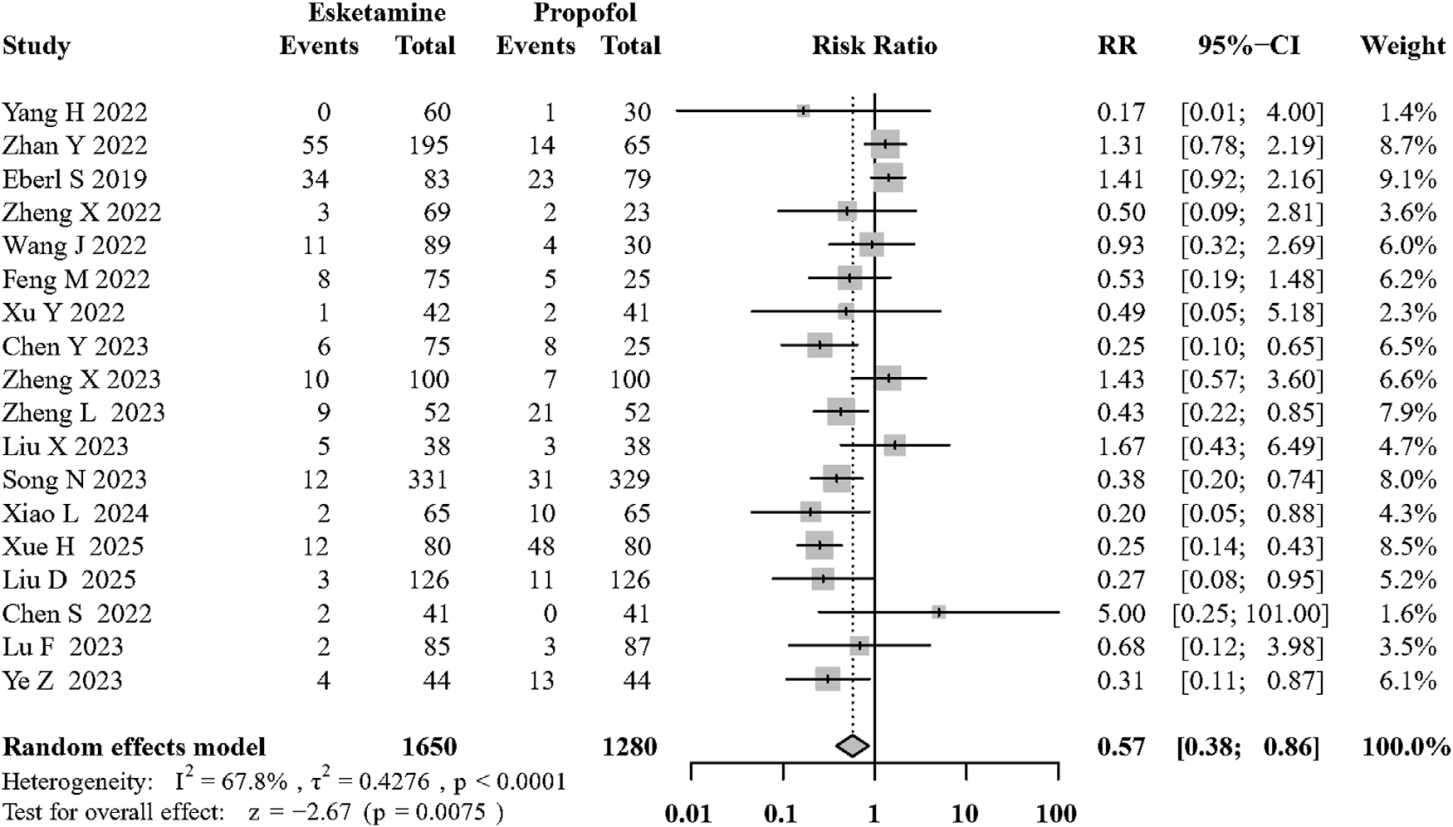
Forest plot of the incidence of adverse respiratory events.

Eighteen of the included RCTs (Yang et al., 2022a; Zhan et al., 2022; Chen et al., 2023a; Feng et al., 2022; Wang et al., 2022; Liu et al., 2023; Zheng et al., 2022; Eberl et al., 2020; Song et al., 2022; Xu et al., 2022; Zheng et al., 2023a; Lu et al., 2023; Chen et al., 2022b; Ye et al., 2023; Liu et al., 2025; Xiao et al., 2024; Xue et al., 2025; Li et al., 2025) evaluated its effects on adverse respiratory events during sedation. Compared with the control group, the use of intravenous esketamine significantly decreased the occurrence of adverse respiratory events (RR: 0.57; 95% CI:0.38 to 0.86, p < 0.01, I^2^ = 67.8%, moderate quality; Figure 4; Supplementary Table S1). The funnel plot and egger’s test did not show significant asymmetry among the included RCTs (Supplementary Figure S1B).

#### Involuntary movement

**FIGURE 5:**
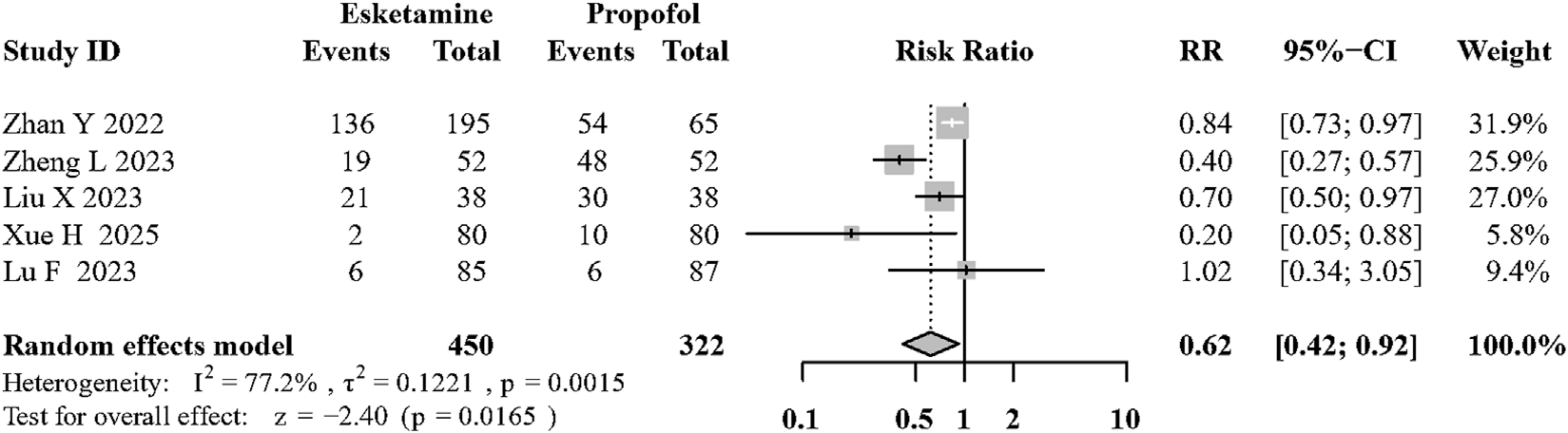
Forest plot of the incidence of involuntary movement during sedation.

Five articles (Zhan et al., 2022; Liu et al., 2023; Lu et al., 2023; Xue et al., 2025; Li et al., 2025) showed the incidence of involuntary body movement during surgery procedures. Esketamine combined with propofol can significantly reduce involuntary body movement compared to propofol based sedation (RR:0.62, 95%CI: 0.41 to 0.92; P = 0.02; I^2^ = 77.2%; low quality; Figure 5; Supplementary Table S1).

#### Propofol consumption

Nine articles (Chen et al., 2023a; Feng et al., 2022; Wang et al., 2022; Liu et al., 2023; Zheng et al., 2022; Eberl et al., 2020; Xu et al., 2022; Ye et al., 2023; Liu et al., 2025) reported the information of propofol consumption during endoscopic procedures. There is significant difference in propofol consumption when esketamine combined with propofol compared to propofol (MD: −0.94, 95%CI: −1.53 to −0.35 (mg/kg); P < 0.01; I^2^ = 96.2%; low quality; Supplementary Figure S11; Supplementary Table S1).

#### Intraoperative hypertension, and arrhythmias during sedation

Five (Zhan et al., 2022; Chen et al., 2023a; Liu et al., 2023; Eberl et al., 2020; Xiao et al., 2024) assessed the occurrence of intraoperative hypertension, and Eleven (Yang et al., 2022a; Chen et al., 2023a; Liu et al., 2023; Eberl et al., 2020; Xu et al., 2022; Lu et al., 2023; Chen et al., 2022b; Ye et al., 2023; Liu et al., 2025; Xiao et al., 2024; Li et al., 2025) evaluated the frequency of intraoperative arrhythmias. There was no difference in the occurrence of intraoperative hypertension (RR: 1.19; 95% CI: 0.61 to 2.33, p = 0.61, I^2^ = 12.8%, moderate quality; Supplementary Figure S12; Supplementary Table S1) and intraoperative arrhythmia (RR: 0.87; 95% CI: 0.48 to 1.59, p = 0.66, I^2^ = 59.2%, moderate quality; Supplementary Figure S13; Supplementary Table S1) between those that received esketamine and the control group.

#### PONV

Thirteen articles (Yang et al., 2022a; Feng et al., 2022; Wang et al., 2022; Zheng et al., 2022; Song et al., 2022; Xu et al., 2022; Zheng et al., 2023a; Lu et al., 2023; Chen et al., 2022b; Ye et al., 2023; Liu et al., 2025; Xiao et al., 2024; Xue et al., 2025) investigated the occurrence of PONV. Compared to the propofol group, patients receiving esketamine did not improve the frequency of PONV (RR: 0.82; 95% CI: 0.44 to 1.53, p = 0.54, I^2^ = 9%, high quality; Supplementary Figure S14; Supplementary Table S1).

#### Postoperative recovery time

Eleven RCTs (Zhan et al., 2022; Chen et al., 2023a; Feng et al., 2022; Wang et al., 2022; Liu et al., 2023; Eberl et al., 2020; Xu et al., 2022; Lu et al., 2023; Chen et al., 2022b; Ye et al., 2023; Li et al., 2025) provided sufficient information about postoperative recovery times, No significant differences in postoperative recovery times were observed between those who did and did not receive esketamine (mean difference: 0.68; 95% CI: −0.71 to 2.07, p = 0.34, I^2^ = 92%, very low quality; Supplementary Figure S15; Supplementary Table S1).

#### Dizziness after awakening

Eleven articles (Zhan et al., 2022; Feng et al., 2022; Wang et al., 2022; Liu et al., 2023; Zheng et al., 2022; Song et al., 2022; Zheng et al., 2023a; Lu et al., 2023; Ye et al., 2023; Xiao et al., 2024; Xue et al., 2025) assessed dizziness after awakening. Patients receiving esketamine might show no significant difference in incidence of dizziness (RR: 1.18; 95% CI: 0.95 to 1.48, p = 0.13, I^2^ = 10.4%, high quality; Supplementary Figure S16; Supplementary Table S1).

### Subgroup analysis and meta-regression analysis

Based on prior empirical observations, subgroup analyses were performed to evaluate the effects of concomitant opioid use (Supplementary Figure S6), including ASA III patients (Supplementary Figure S7), age >18 years (Supplementary Figure S8), the dose of esketamine (≤0.2 mg vs. >0.2 mg/kg) (Supplementary Figure S9) and surgical location (Supplementary Figure S10) on primary outcome measures. Meanwhile, we found significant heterogeneity between different subgroups. All results indicated that while the incidence of hypotension remained consistent across these subgroups (preserving effect sizes), these stratification criteria effectively reduced inter-subgroup heterogeneity levels.

Additionally, meta-regression incorporating sample size and mean patient age was performed to assess all study outcomes (Supplementary Table S2; Supplementary Table S3). This analysis demonstrated a significant association between sample size and the effect size of recovery time (β = 0.03, 95% CI: 0.02 to 0.05, p < 0.01, Supplementary Table S2), revealing a dose-response relationship where larger sample sizes enhanced the superiority of esketamine combined with propofol in accelerating recovery. A bubble plot was subsequently generated to visually demonstrate this dose-response relationship through weighted regression modeling (Supplementary Figure S17).

## Discussion

This meta-analysis synthesized evidence from 18 randomized controlled trials involving 2,930 participants to evaluate esketamine as an adjunct to propofol-based sedation during gastrointestinal endoscopy. Our findings demonstrate that adding intravenous esketamine: 1) reduced the risk of intra-procedural hypotension by 68% (RR: 0.32), 2) decreased adverse respiratory events by 43% (RR: 0.57), 3) reduced involuntary movement and propofol consumption, while 4) not increasing hypertension, arrhythmic, recovery time, or dizziness. These results suggest that esketamine can counterbalance propofol’s cardiovascular depressive effects while maintaining an acceptable safety profile.

Previous meta-analysis discussed the efficacy of esketamine in acute postoperative pain reduction in elective surgery (Brinck et al., 2021) and the safety and efficacy of esketamine in procedural sedation analgesia (Lian et al., 2023; Huang et al., 2023b). Compared with these studies, the novel points of our study were: 1) we comprehensively explored the effect of esketamine on intraoperative hemodynamic and respiratory parameters, which are the most worrying side effect related to sedation, whereas the previous study focused on patients’ recovery and propofol consumption (Lian et al., 2023). It is important to emphasize the effect of esketamine on hypotension because propofol has outstanding side effect of hypotension; 2) This study specifically compared esketamine-propofol combination therapy against propofol. This head-to-head comparison minimizes confounding from heterogeneous drug regimens while maintaining methodological rigor in intervention design.

Propofol exerts dose-dependent inhibitory effects on cardiovascular and respiratory systems, particularly with opioids, prompting clinical research toward novel drugs or optimized combinations for procedural sedation to reduce adverse effects while preserving efficacy (Hug et al., 1993; Nieuwenhuijs et al., 2001; Chen et al., 2023b). In our experiment, compared to the control group using propofol alone, the addition of esketamine resulted in a reduction of approximately 1 mg of propofol per kilogram of body weight. This effect contributed to the stabilization of hemodynamics and respiratory mechanics during the combination therapy, reducing the incidence of airway intervention events, lowering the need for high-level staffing in case of emergencies and other perioperative adverse events. Although there is currently a lack of RCTs specifically targeting obese, elderly, and high-risk patients, our study primarily focused on ASA Ⅰ-Ⅲ, and normal BMI. However, limited studies in elderly, high-risk, and overweight populations still suggest that esketamine can effectively reduce perioperative complications during sedation or surgical procedures (Zhang et al., 2025; Zheng et al., 2023b; Li et al., 2025).

Esketamine shares the same basic properties of ketamine; however, its anesthetic effect is about two times that of racemic ketamine, with fewer psychomimetic effects and a rapid recovery from anesthesia (Krys et al., 1994; Wang et al., 2019). In addition to its NMDA antagonism, which provides analgesia, preserved airway reflexes and catecholamine release help limit respiratory and cardiovascular suppression. However, the psychological effects should not be overlooked, as dizziness and dissociative symptoms have been reported in several clinical studies. Although these effects are typically minimal due to single low-dose administration in painless procedures, their potential impact should still be considered (Hirota and Lambert, 2022; Akin et al., 2005; Schultetus et al., 1985). Compared to opioid anesthetics, applying esketamine to gastrointestinal endoscopy sedation may be safer for its hemodynamic and respiratory benefit, and not increase the incidence of dizziness (Mion and Villevieille, 2013; Engelhardt et al., 1998). Although the use of esketamine for procedural sedation remains off-label and is not currently endorsed by international guidelines, emerging evidence supports its exploratory application in this context. Nonetheless, further large-scale, high-quality trials are necessary before it can be recommended for routine clinical practice.

Our meta-analysis has several important limitations that affect the interpretation and clinical application of our findings. First, Substantial statistical heterogeneity was observed across most outcomes (I^2^>50%), limiting the reliability of our pooled estimates. This heterogeneity likely stems from multiple sources including variations in control group composition (propofol alone vs. propofol with adjuvants), esketamine dosing regimens (0.1–0.5 mg/kg), patient populations, and procedural types. Subgroup analyses based on concomitant opioid use, esketamine dosing, and procedure type provided some explanation for heterogeneity but did not eliminate it entirely. Therefore, our findings should be interpreted cautiously given this substantial between-study variation. Second, the funnel plot for the primary outcome showed asymmetry, suggesting potential publication bias or small-study effects. This asymmetry may result from unpublished smaller studies with null findings, variations in study methodology, or genuine clinical heterogeneity across included trials. Although the trim-and-fill method suggests our primary conclusion is robust to potential publication bias, readers should interpret our findings with appropriate caution given this limitation. Third, while our analysis suggests no significant increase in dizziness, the included studies did not adequately evaluate patients’ ability to perform activities of daily living (such as driving, procedure satisfaction or other procedural tolerability) on the day following the procedure. This represents a critical gap in patient counseling information and warrants investigation in future studies designed with patient-centered outcomes. Fourth, while our analysis demonstrates significant hemodynamic benefits of esketamine, another limitation is the lack of comprehensive time-course data across included studies. Most trials reported hemodynamic parameters at discrete time points rather than providing detailed temporal analysis. Future studies should incorporate systematic time-resolved monitoring to better characterize the dynamic nature of hemodynamic responses to esketamine-propofol combinations.

## Conclusion

Adding intravenous esketamine to propofol-based sedation for gastrointestinal endoscopy improves hemodynamic stability and enhances respiratory safety. Given the moderate-certainty evidence, larger rigorously designed trials are needed to confirm these benefits, optimize dosing, and clarify neurosensory safety.

## Data Availability

The raw data supporting the conclusions of this article will be made available by the authors, without undue reservation.
