# Supplementary material for: Adjunctive esketamine in propofol-based sedation for gastrointestinal endoscopy: a systematic review and meta-analysis of randomized trials
Source: Front Pharmacol. 2025 Nov 25;16:1662057. doi: 10.3389/fphar.2025.1662057 (PMC12685882; doi:10.3389/fphar.2025.1662057)
Supplement: Supplementary file 1 [file DataSheet1.docx]

**Supplementary files provided by authors to supplement the manuscript entitled**

**“Adjunctive Esketamine in Propofol‑Based Sedation for Gastrointestinal Endoscopy: A systematic review and meta-analysis of randomized trials”**

**Content**

- **Supplementary Table I. Quality of evidence for primary outcome and secondary outcome.**

[Page3-6]

- **Supplementary Table II. Meta-regression assesses the impact of sample size on: hypotension; respiratory adverse events; propofol consumption; involuntary movement; hypertension; arrhythmia; PONV; recovery time and dizziness.**

[Page 7-8]

- **Supplementary Table III. Meta-regression assesses the impact of mean age on: hypotension; respiratory adverse events; propofol consumption; involuntary movement; hypertension; arrhythmia; PONV; recovery time and dizziness.**

[Page 9-10]

- **Supplementary Figure 1.** **Funnel plot of outcomes.**

[Page 11-12]

- **Supplementary Figure 2. “Leave-one-out” sensitivity analysis of the incidence of intraoperative hypotension.**

[Page 13]

- **Supplementary Figure 3. Trim-and-Fill adjusted forest plots for** **the incidence of intraoperative hypotension.**

[Page 14]

- **Supplementary Figure 4. Trim-and-Fill adjusted funnel plots for** **the incidence of intraoperative hypotension.**

[Page 15]

- **Supplementary Figure 5. Forest plot of the incidence of hypotension after exclude high risk bias studies.**

[Page 16]

- **Supplementary Figure 6. The subgroup analysis of the incidence of hypotension related to Opioid.**

[Page17]

- **Supplementary Figure 7. The subgroup analysis of the incidence of hypotension related to ASA class.**

[Page 18]

- **Supplementary Figure 8. The subgroup analysis of the incidence of hypotension related to age.**

[Page 19]

- **Supplementary Figure 9. The subgroup analysis of the incidence of hypotension related to the dose of esketamine(≤0.2 mg/kg vs. >0.2 mg/kg).**

[Page 20]

- **Supplementary Figure 10. The subgroup analysis of the incidence of hypotension related to surgical location.**

[Page 21]

- **Supplementary Figure 11. Forest plot of the propofol consumption (mg/kg).**

[Page 22]

- **Supplementary Figure 12. Forest plot of the incidence of hypertension.**

[Page 23]

- **Supplementary Figure 13. Forest plot of the incidence of arrhythmia.**

[Page 24]

- **Supplementary Figure 14. Forest plot of the incidence of PONV.**

[Page 25]

- **Supplementary Figure 15. Forest plot of recovery time after surgery.**

[Page 26]

- **Supplementary Figure 16. Forest plot of the incidence of dizziness after surgery.**

[Page 27]

- **Supplementary Figure 17. Bubble Patterns in Meta-Regression: Sample Size Impacts on Surgical Recovery Time.**

[Page 28]

- **Supplementary Figure 18. Forest plot of mean arterial pressure between the two groups after induction.**

[Page 29]

- **Appendix. 1 Search strategies.**

[Page 30-33]

- **References**
  [Page 34]

**Supplementary Table I. Quality of evidence for primary outcome and secondary outcome.**

| **Certainty assessment** | | | | | | | **№ of patients** | | **Effect** | | **Certainty** |
| --- | --- | --- | --- | --- | --- | --- | --- | --- | --- | --- | --- |
| **№ of studies** | **Study design** | **Risk of bias** | **Inconsistency** | **Indirectness** | **Imprecision** | **Other considerations** | **Esketamine** | **Propofol‑** | **Relative (95% CI)** | **Absolute (95% CI)** |  |
| **Hypotension^a^** | | | | | | | | | | | |
| 16 | randomised trials | not serious | not serious | not serious | not serious | publication bias strongly suspected^b^ | 127/1509 (8.4%) | 280/1139 (24.6%) | **RR 0.32** (0.24 to 0.43) | **17 fewer per 100** (from 19 fewer to 14 fewer) | ⨁⨁⨁◯ Moderate^b^ |
| **Respiratory adverse events^c^** | | | | | | | | | | | |
| 18 | randomised trials | not serious | serious^d^ | not serious | not serious | none | 179/1650 (10.8%) | 206/1280 (16.1%) | **RR 0.57** (0.38 to 0.86) | **69 fewer per 1,000** (from 100 fewer to 23 fewer) | ⨁⨁⨁◯ Moderate^d^ |
| **Propofol consumption^e^** | | | | | | | | | | | |
| 9 | randomised trials | not serious | serious^d^ | not serious | not serious | publication bias strongly suspected^f^ | 641 | 431 | - | MD **0.94 mg/kg fewer** (1.53 fewer to 0.35 fewer) | ⨁⨁◯◯ Low^d,f^ |
| **Involuntary movement^g^** | | | | | | | | | | | |
| 5 | randomised trials | not serious | serious^d^ | not serious | not serious | publication bias strongly suspected^f^ | 184/450 (40.9%) | 148/322 (46.0%) | **RR 0.61** (0.42 to 0.92) | **179 fewer per 1,000** (from 267 fewer to 37 fewer) | ⨁⨁◯◯ Low^d,f^ |
| **Hypertension^h^** | | | | | | | | | | | |
| 5 | randomised trials | not serious | not serious | not serious | not serious | publication bias strongly suspected^f^ | 37/456 (8.1%) | 20/272 (7.4%) | **RR 1.19** (0.61 to 2.33) | **14 more per 1,000** (from 29 fewer to 98 more) | ⨁⨁⨁◯ Moderate^f^ |
| **Arrhythmia^i^** | | | | | | | | | | | |
| 11 | randomised trials | not serious | serious^j^ | not serious | not serious | none | 87/711 (12.2%) | 70/628 (11.1%) | **RR 0.87** (0.48 to 1.59) | **14 fewer per 1,000** (from 58 fewer to 66 more) | ⨁⨁⨁◯ Moderate^j^ |
| **PONV^k^** | | | | | | | | | | | |
| 13 | randomised trials | not serious | not serious | not serious | not serious | none | 26/1207 (2.2%) | 30/1021 (2.9%) | **RR 0.82** (0.44 to 1.53) | **5 fewer per 1,000** (from 16 fewer to 16 more) | ⨁⨁⨁⨁ High |
| **Recovery time^l^** | | | | | | | | | | | |
| 11 | randomised trials | not serious | very serious^m^ | not serious | serious^n^ | none | 819 | 527 | - | MD **0.68 min higher** (0.71 lower to 2.07 higher) | ⨁◯◯◯ Very low^m,n^ |
| **Dizziness** | | | | | | | | | | | |
| 11 | randomised trials | not serious | not serious | not serious | not serious | none | 221/1171 (18.9%) | 138/886 (15.6%) | **RR 1.18** (0.95 to 1.48) | **28 more per 1,000** (from 8 fewer to 75 more) | ⨁⨁⨁⨁ High |

**CI:** confidence interval; **MD:** mean difference; **RR:** risk ratio

* The sample size is the sum of patients included in the body of evidence.

† Required information size was evaluated according to GRADE guidelines 6.[1]

‡ The mean difference, its 95% CI, and I^2^ were calculated based on the pairwise meta-analysis.

§ Calculated as the number of studies with high ROB divided by the number of studies in the body of evidence. The details of the assessment of ROB are presented in figure SF1.

¶ The evidence quality was graded according to the GRADE guidelines.[2]

**Explanations**

a. Hypotension was defined as a decrease in mean arterial pressure from baseline of more than 20% and systolic blood pressure below 80 mmHg or 80% of the baseline value, as previously described[3, 4].

b. Downgraded one level for publication bias. The publication bias was determined by visual inspection of the funnel plots, with asymmetry suggesting the existence of publication bias.

c. Respiratory adverse events, which was defined as hypoxemia (oxygen saturation level below 95% based on pulse oximetry) lasting longer than 10s, laryngospasm, increased oxygen flow, asphyxia, and the requirement of mechanical ventilation during surgery[5, 6].

d. Downgraded one level for inconsistency as a result of high statistical heterogeneity (I^2^ ≥ 60%) that could not be explained by subgroup analyses[7].

e. Propofol consumption was defined as the amount of propofol consumed per kilogram of body weight throughout the sedation process[8, 9].

f. The funnel plot shows asymmetry, suggesting the presence of publication bias.

g. Involuntary movement refers to unconscious muscle contractions or limb movements triggered by physiological reflexes (such as pharyngeal reflex or intestinal spasm) or painful stimuli during the examination[10].

h. Hypertension was defined as blood pressure 30% higher than baseline values. Arrhythmias included bradycardia, tachycardia, or other types of irregular heartbeat[11].

i. Arrhythmia refers to the abnormal rhythm or heart rate of the heart, which mainly includes tachycardia, bradycardia, and abnormalities in the location of occurrence.

j. The heterogeneity is substantial, with the actual heterogeneity (I²) being 59.2%.

k. Postoperative nausea and vomiting (PONV) refers to the symptoms of nausea or vomiting that occur in patients after surgery, caused by the effects of anesthesia, the surgical procedure, medications, or other factors.

l. Postoperative recovery time was defined as the length of time required for a full restoration of consciousness after sedation/anesthesia, an Observer Assessment of Alertness/Sedation (OAA/S) score greater than 5, or a modified OAA/S score greater than 4[12].

m. The heterogeneity is substantial, with the actual heterogeneity (I²) being 92.0%.

n. Downgraded one level for imprecision because the required information size was not met, or the prediction interval is wide[1].

**Supplementary Table II. Meta-regression assesses the impact of sample size on: hypotension; respiratory adverse events; hypertension; arrhythmia; PONV; endoscopist satisfaction; recovery time and dizziness.**

| Hypotension | Effect estimate | *P*-value | R^2^ | I^2^ | Test for residual heterogeneity |
| --- | --- | --- | --- | --- | --- |
| Intrcpt | -1.2052 | <0.0001 | 0 | 48.07% | 0.0195 |
| Sample size | 0.0002 | 0.8249 |  |  |  |

| respiratory adverse events | Effect estimate | *P*-value | R^2^ | I^2^ | Test for residual heterogeneity |
| --- | --- | --- | --- | --- | --- |
| Intrcpt | -0.4983 | 0.1475 | 0 | 69.53% | <0.0001 |
| Sample size | -0.0003 | 0.8262 |  |  |  |

| Propofol consumption | Effect estimate | *P*-value | R^2^ | I^2^ | Test for residual heterogeneity |
| --- | --- | --- | --- | --- | --- |
| Intrcpt | -0.8199 | 0.2976 | 0 | 97.53% | < 0.0001 |
| Sample size | -0.0011 | 0.8613 |  |  |  |

| Involuntary movement | Effect estimate | *P*-value | R^2^ | I^2^ | Test for residual heterogeneity |
| --- | --- | --- | --- | --- | --- |
| Intrcpt | -0.8610 | 0.1001 | 0 | 68.27% | 0.0238 |
| Sample size | 0.0024 | 0.4369 |  |  |  |

| Hypertension | Effect estimate | *P*-value | R^2^ | I^2^ | Test for residual heterogeneity |
| --- | --- | --- | --- | --- | --- |
| Intrcpt | 1.4335 | 0.3007 | 0 | 17.56% | 0.3032 |
| Sample size | -0.0084 | 0.3643 |  |  |  |

| arrhythmia | Effect estimate | *P*-value | R^2^ | I^2^ | Test for residual heterogeneity |
| --- | --- | --- | --- | --- | --- |
| Intrcpt | 0.9295 | 0.1593 | 37.86% | 44.81% | 0.0607 |
| Sample size | -0.0078 | 0.0868 |  |  |  |

| PONV | Effect estimate | *P*-value | R^2^ | I^2^ | Test for residual heterogeneity |
| --- | --- | --- | --- | --- | --- |
| Intrcpt | -0.4266 | 0.3904 | 0 | 13.90% | 0.3082 |
| Sample size | 0.0009 | 0.5524 |  |  |  |

| recovery time | Effect estimate | *P*-value | R^2^ | I^2^ | Test for residual heterogeneity |
| --- | --- | --- | --- | --- | --- |
| Intrcpt | -3.3386 | 0.0005 | 74.08% | 79.93% | <0.0001 |
| Sample size | 0.0324 | <.0001 |  |  |  |

| dizziness | Effect estimate | *P*-value | R^2^ | I^2^ | Test for residual heterogeneity |
| --- | --- | --- | --- | --- | --- |
| Intrcpt | 0.2611 | 0.1674 | 0% | 13.33% | 0.3203 |
| Sample size | -0.0003 | 0.5539 |  |  |  |

**Supplementary Table III. Meta-regression assesses the impact of mean age on: hypotension; respiratory adverse events; hypertension; arrhythmia; PONV; endoscopist satisfaction; recovery time and dizziness.**

| Hypotension | Effect estimate | *P*-value | R^2^ | I^2^ | Test for residual heterogeneity |
| --- | --- | --- | --- | --- | --- |
| Intrcpt | -0.5472 | 0.1758 | 12.82% | 39.28% | 0.0594 |
| Age | -0.0135 | 0.1195 |  |  |  |

| respiratory adverse events | Effect estimate | *P*-value | R^2^ | I^2^ | Test for residual heterogeneity |
| --- | --- | --- | --- | --- | --- |
| Intrcpt | 0.0416 | 0.9460 | 0 | 69.63% | < 0.0001 |
| Age | -0.0140 | 0.2979 |  |  |  |

| Propofol consumption | Effect estimate | *P*-value | R^2^ | I^2^ | Test for residual heterogeneity |
| --- | --- | --- | --- | --- | --- |
| Intrcpt | -1.8596 | 0.0095 | 11.45% | 96.86% | < 0.0001 |
| Age | 0.0216 | 0.1634 |  |  |  |

| Involuntary movement | Effect estimate | *P*-value | R^2^ | I^2^ | Test for residual heterogeneity |
| --- | --- | --- | --- | --- | --- |
| Intrcpt | -4.7672 | 0.13687 | 0 | 77.52% | 0.0040 |
| Age | 0.0949 | 0.1804 |  |  |  |

| Hypertension | Effect estimate | *P*-value | R^2^ | I^2^ | Test for residual heterogeneity |
| --- | --- | --- | --- | --- | --- |
| Intrcpt | 7.6480 | 0.1540 | 100% | 0 | 0.4579 |
| Age | -0.1373 | 0.1585 |  |  |  |

| arrhythmia | Effect estimate | *P*-value | R^2^ | I^2^ | Test for residual heterogeneity |
| --- | --- | --- | --- | --- | --- |
| Intrcpt | 2.1314 | 0.3416 | 5.04% | 57.56% | 0.0118 |
| Age | -0.0434 | 0.3079 |  |  |  |

| PONV | Effect estimate | *P*-value | R^2^ | I^2^ | Test for residual heterogeneity |
| --- | --- | --- | --- | --- | --- |
| Intrcpt | -1.5527 | 0.0965 | 100% | 0% | 0.4617 |
| Age | 0.0316 | 0.1212 |  |  |  |

| recovery time | Effect estimate | *P*-value | R^2^ | I^2^ | Test for residual heterogeneity |
| --- | --- | --- | --- | --- | --- |
| Intrcpt | 4.8927 | 0.2224 | 1.26% | 93.70% | < 0.0001 |
| Age | -0.0882 | 0.2859 |  |  |  |

| dizziness | Effect estimate | *P*-value | R^2^ | I^2^ | Test for residual heterogeneity |
| --- | --- | --- | --- | --- | --- |
| Intrcpt | 0.7914 | 0.0199 | 100% | 0% | 0.6008 |
| Age | -0.0154 | 0.0509 |  |  |  |

**Supplementary Figure 1. Funnel plot of the incidence of outcomes [ hypotension(A); respiratory adverse events(B); propofol consumption(C); Involuntary movement(D); hypertension(E); arrhythmia(F); PONV(J); recovery time(H) and dizziness(I)].**


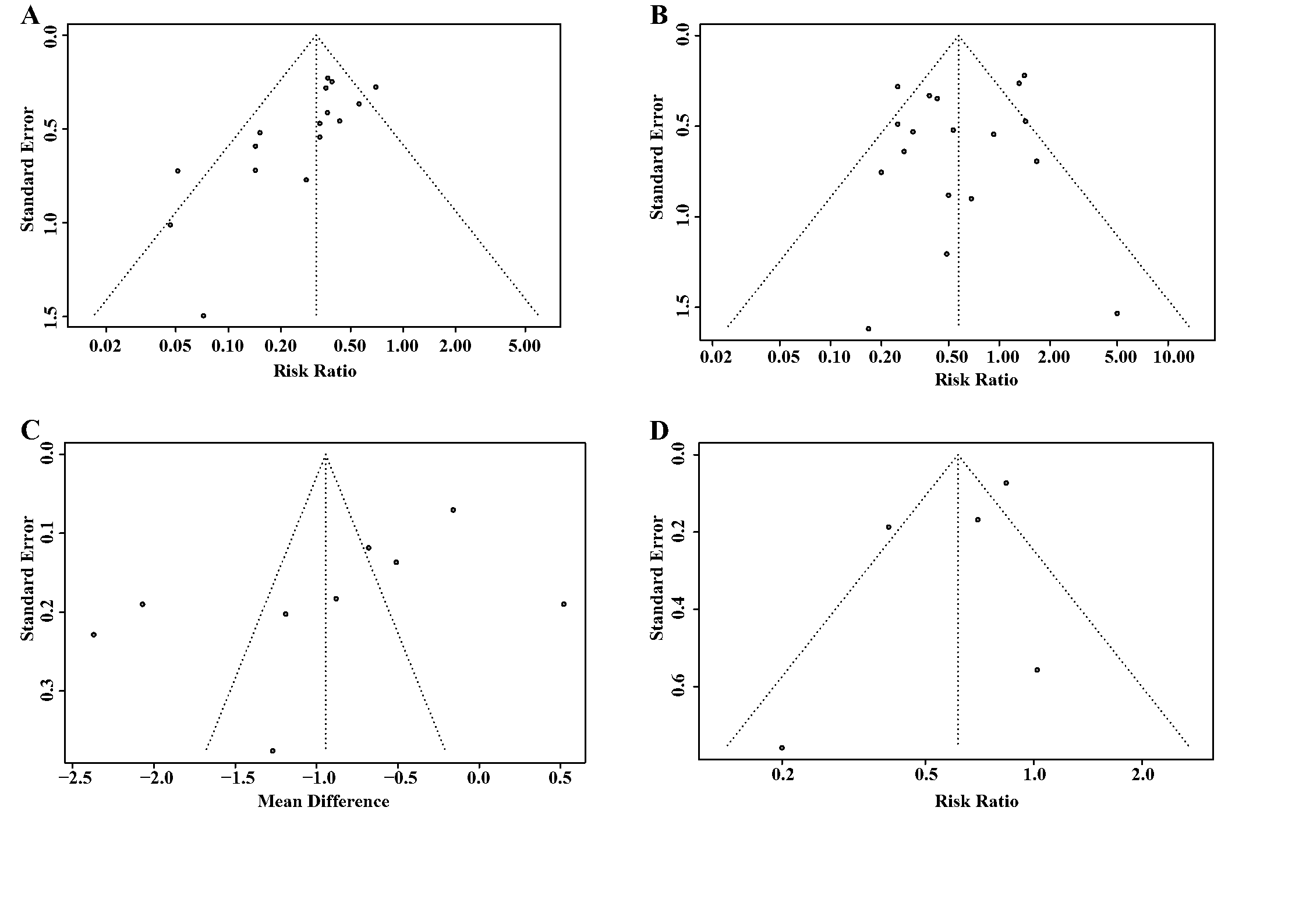


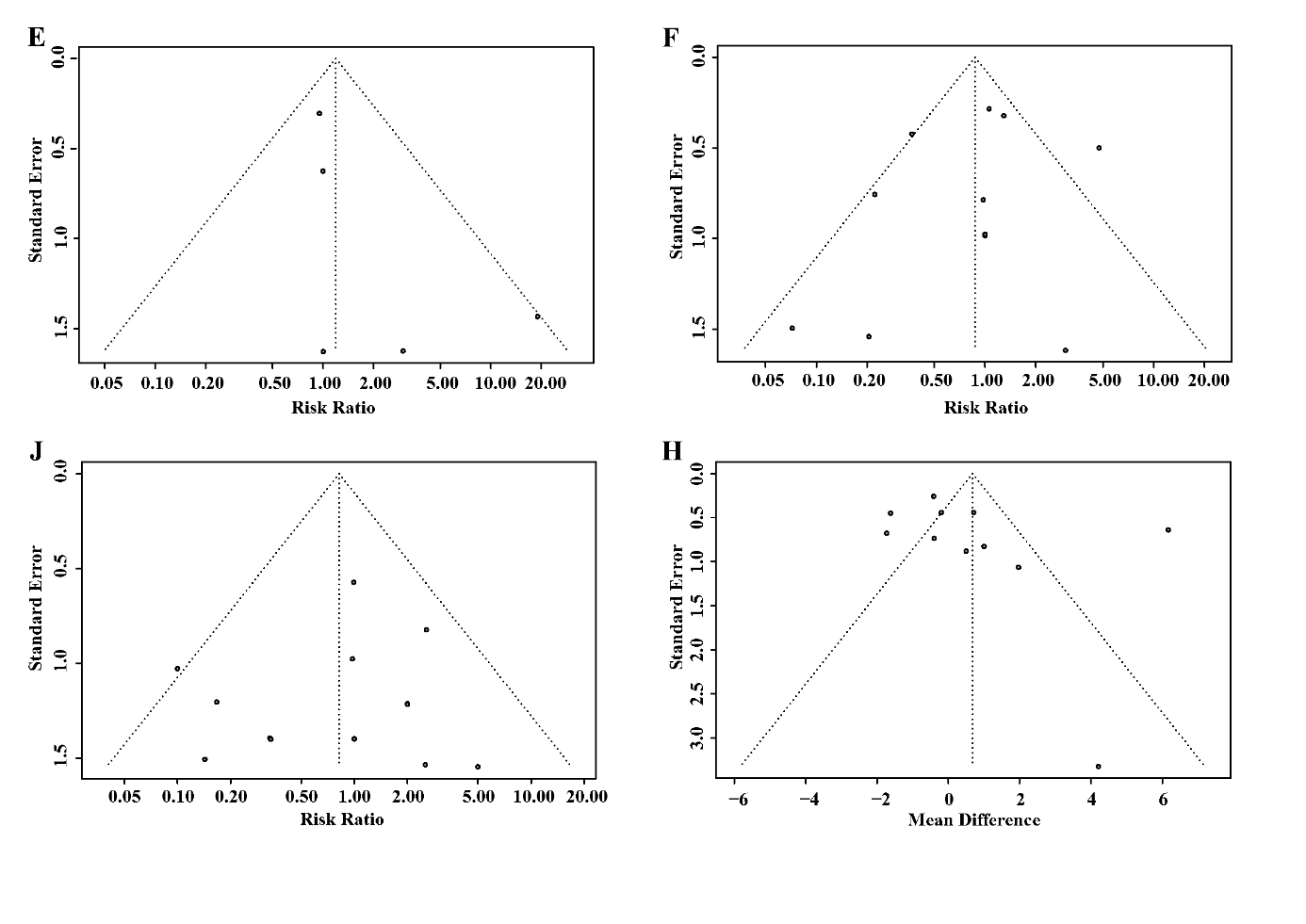


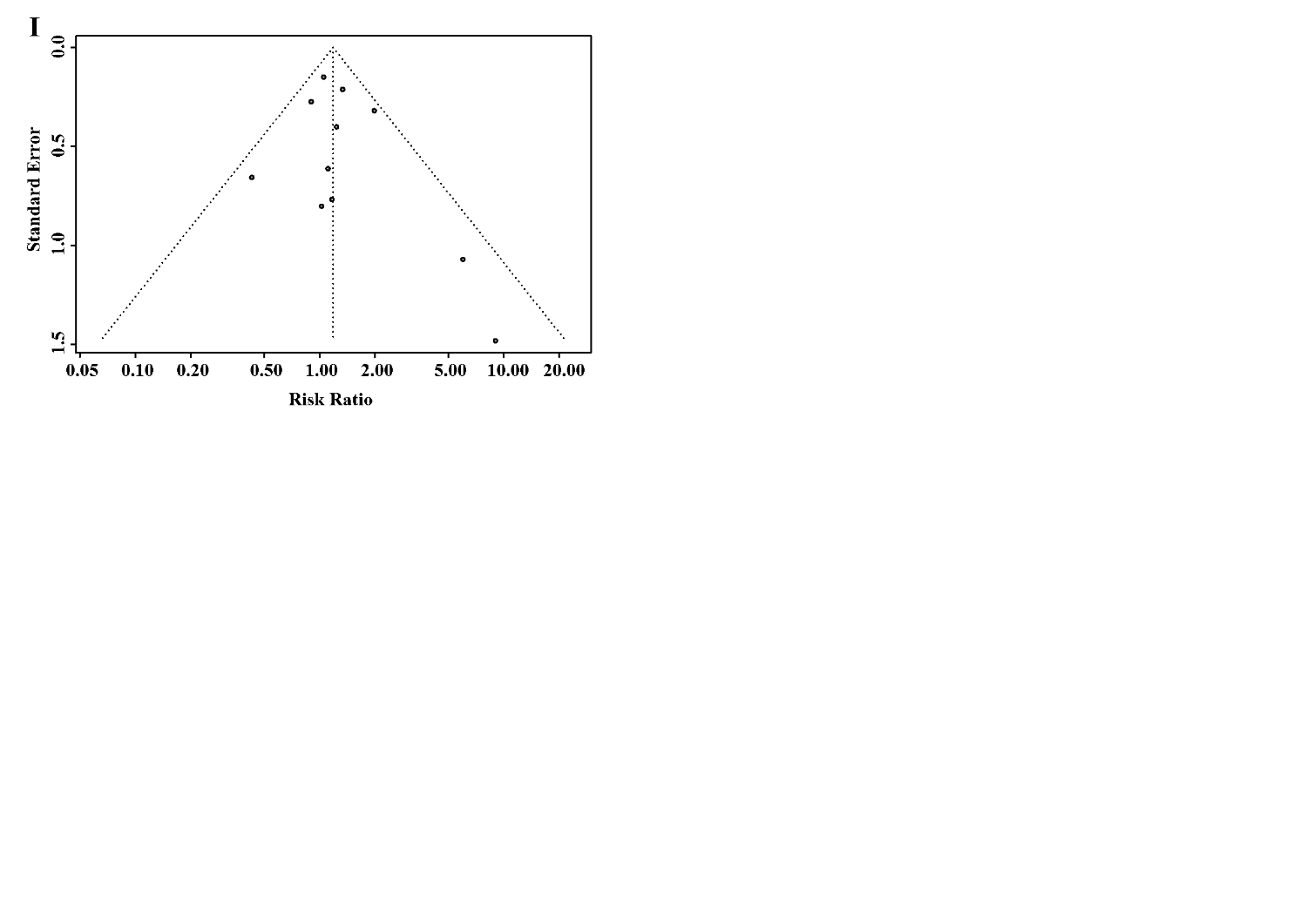


**Supplementary** **Figure 2: “Leave-one-out” sensitivity analysis of the incidence of intraoperative hypotension.**

**
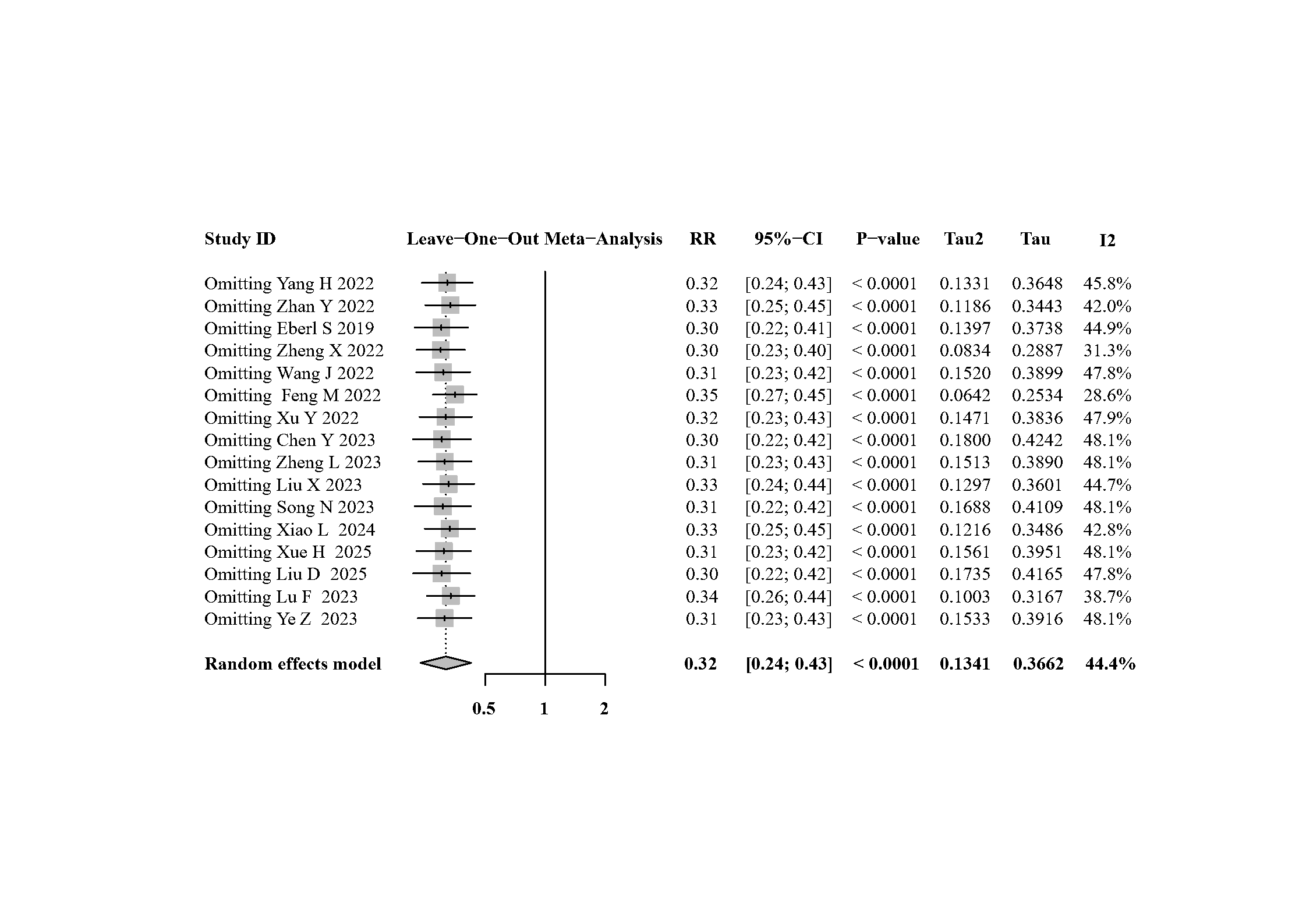
**

**Supplementary Figure 3. Trim-and-Fill adjusted forest plots for** **the incidence of intraoperative hypotension.**

**
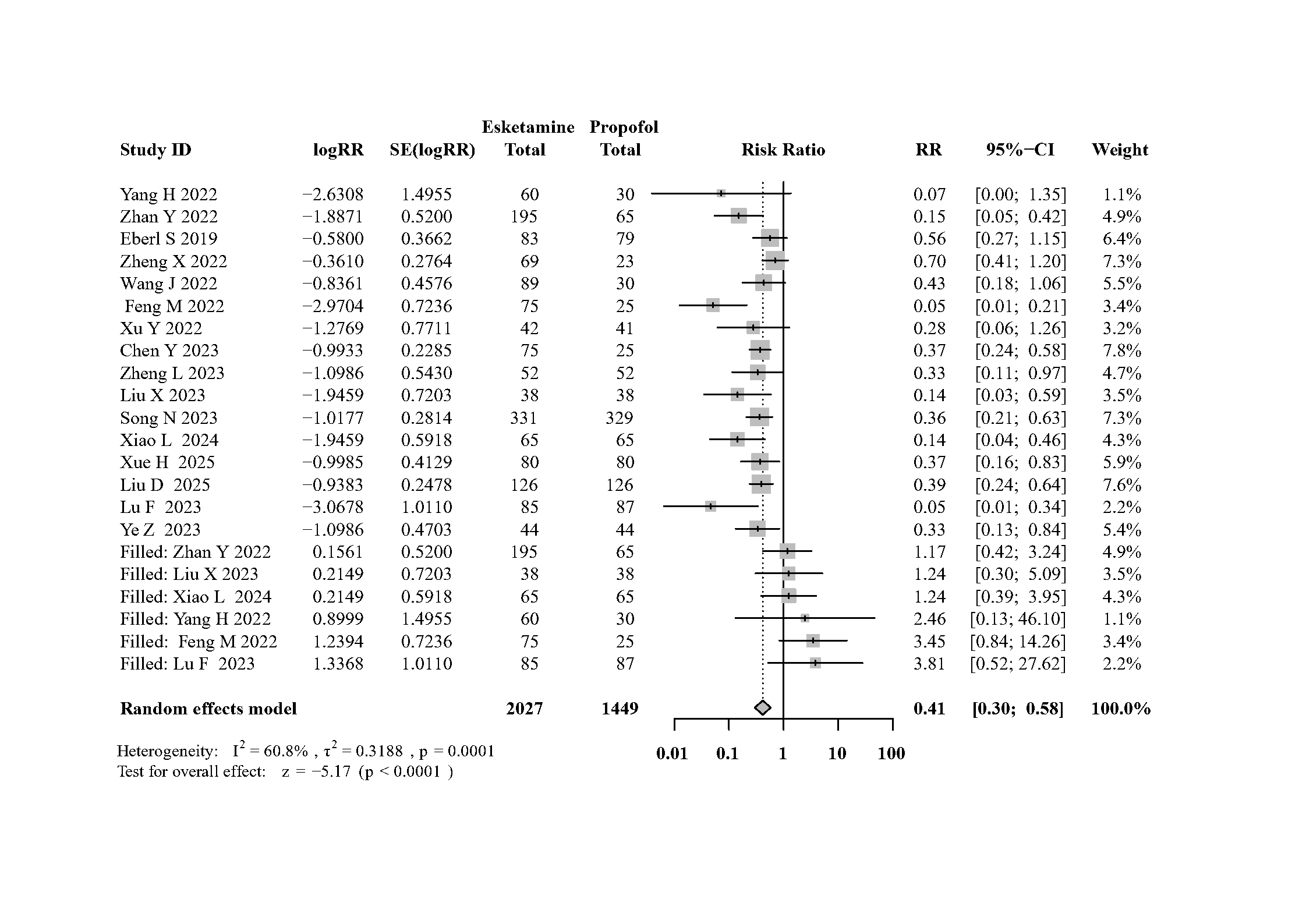
**

**Supplementary Figure 4. Trim-and-Fill adjusted funnel plots for** **the incidence of intraoperative hypotension.**

**
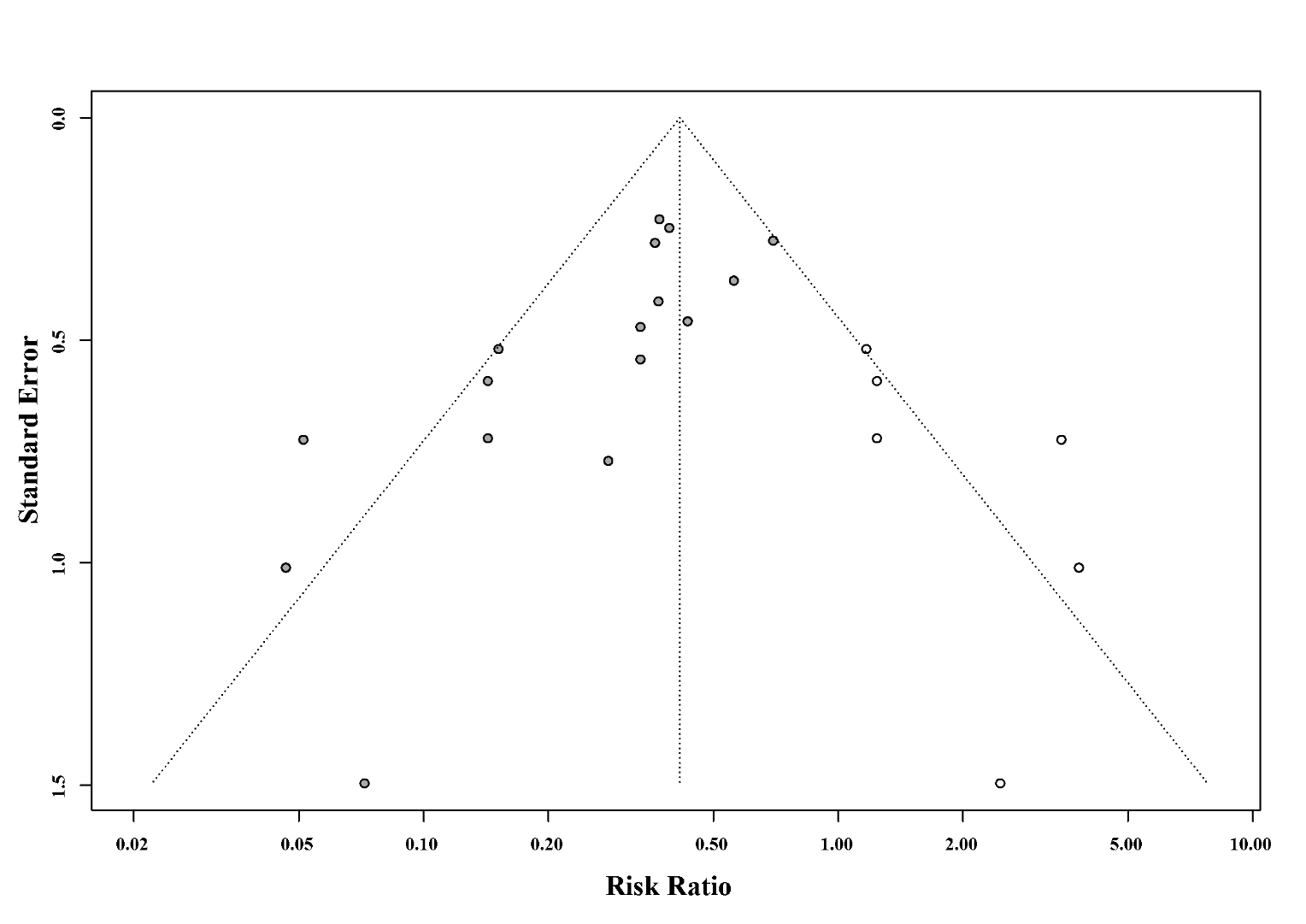
**

**Supplementary Figure 5. Forest plot of the incidence of hypotension after excluding high risk-of-bias studies.**
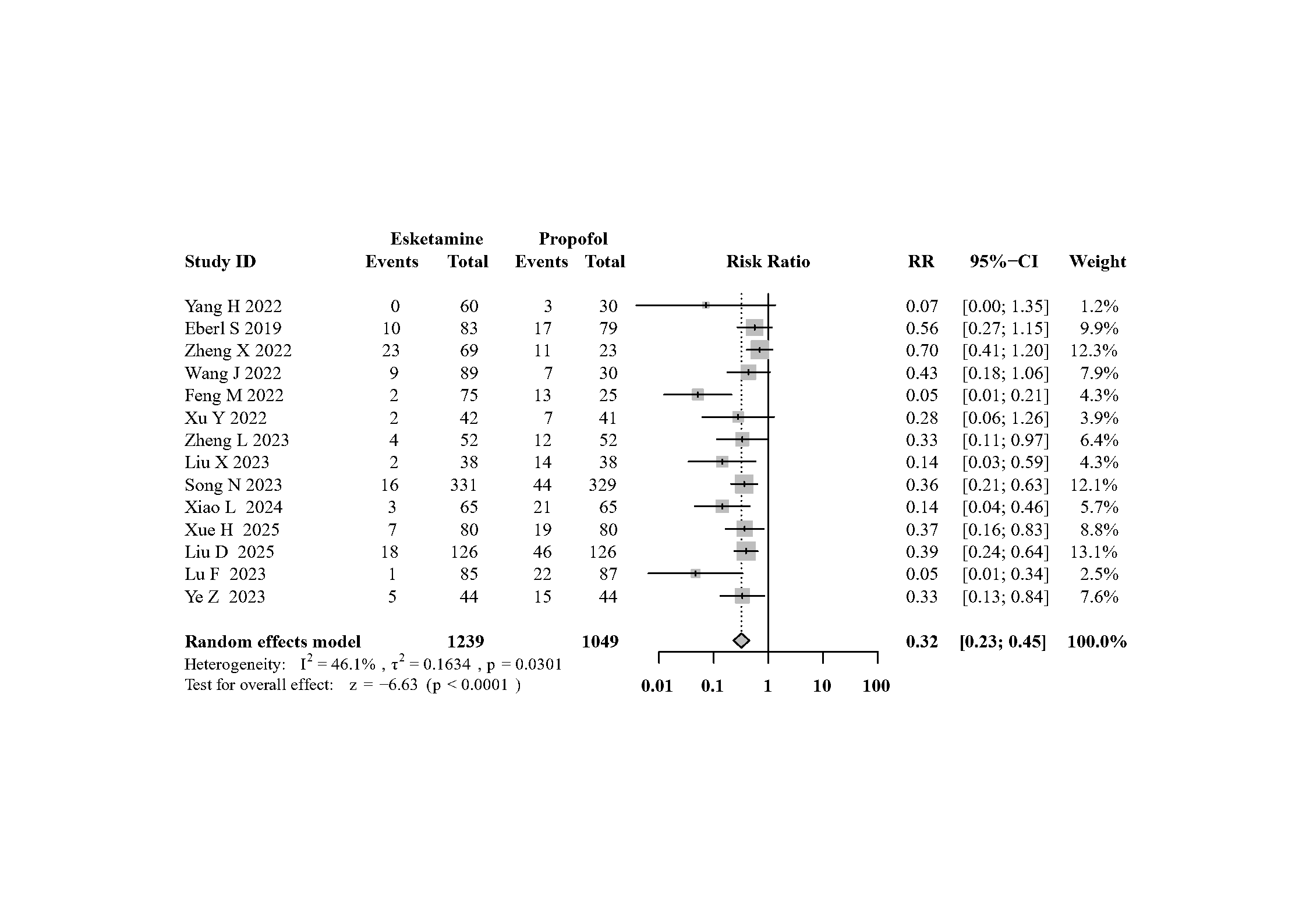


**Supplementary Figure 6. The subgroup analysis of the incidence of hypotension related to Opioid.**


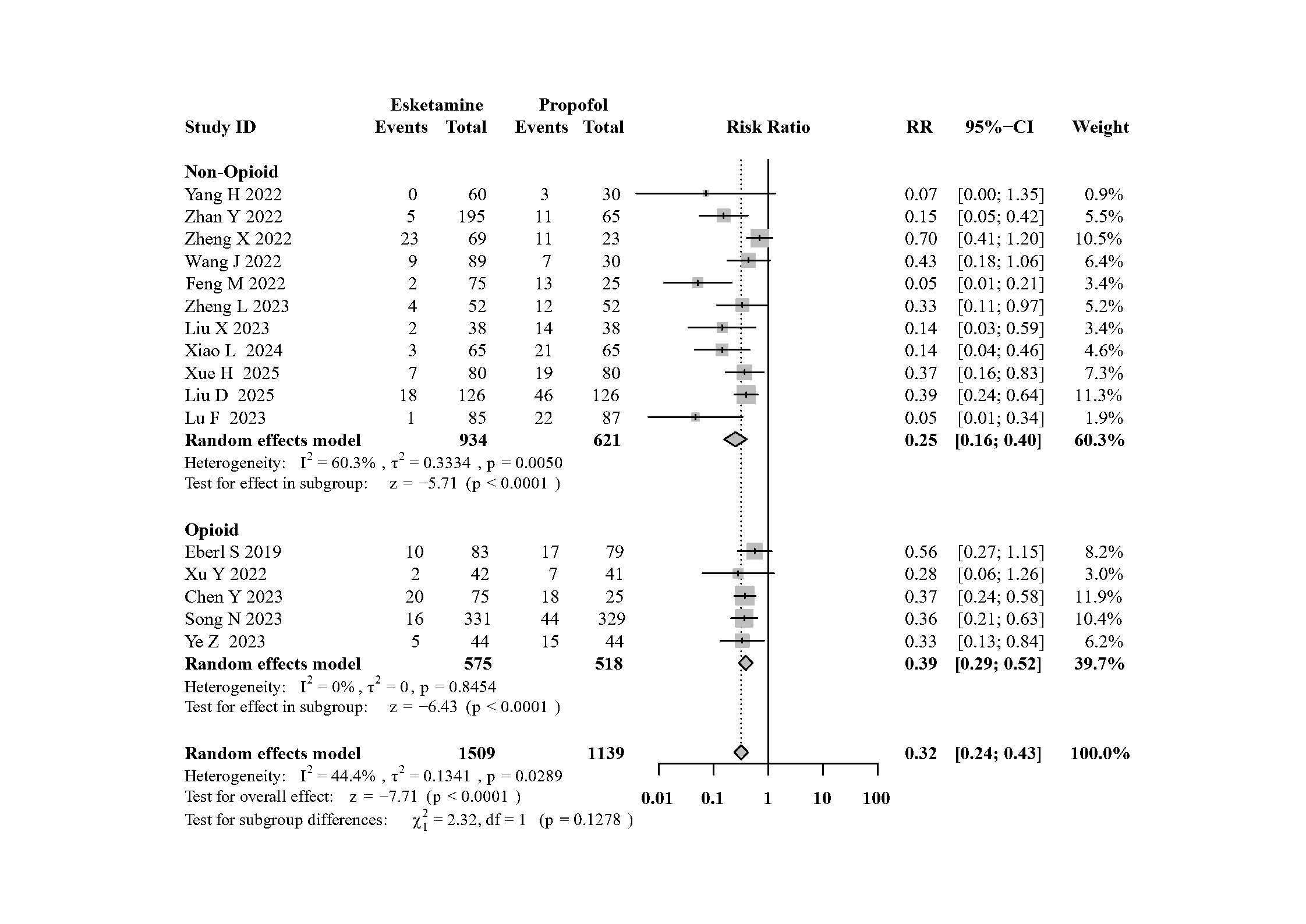


**Supplementary Figure 7. The subgroup analysis of the incidence of hypotension related to ASA class.**


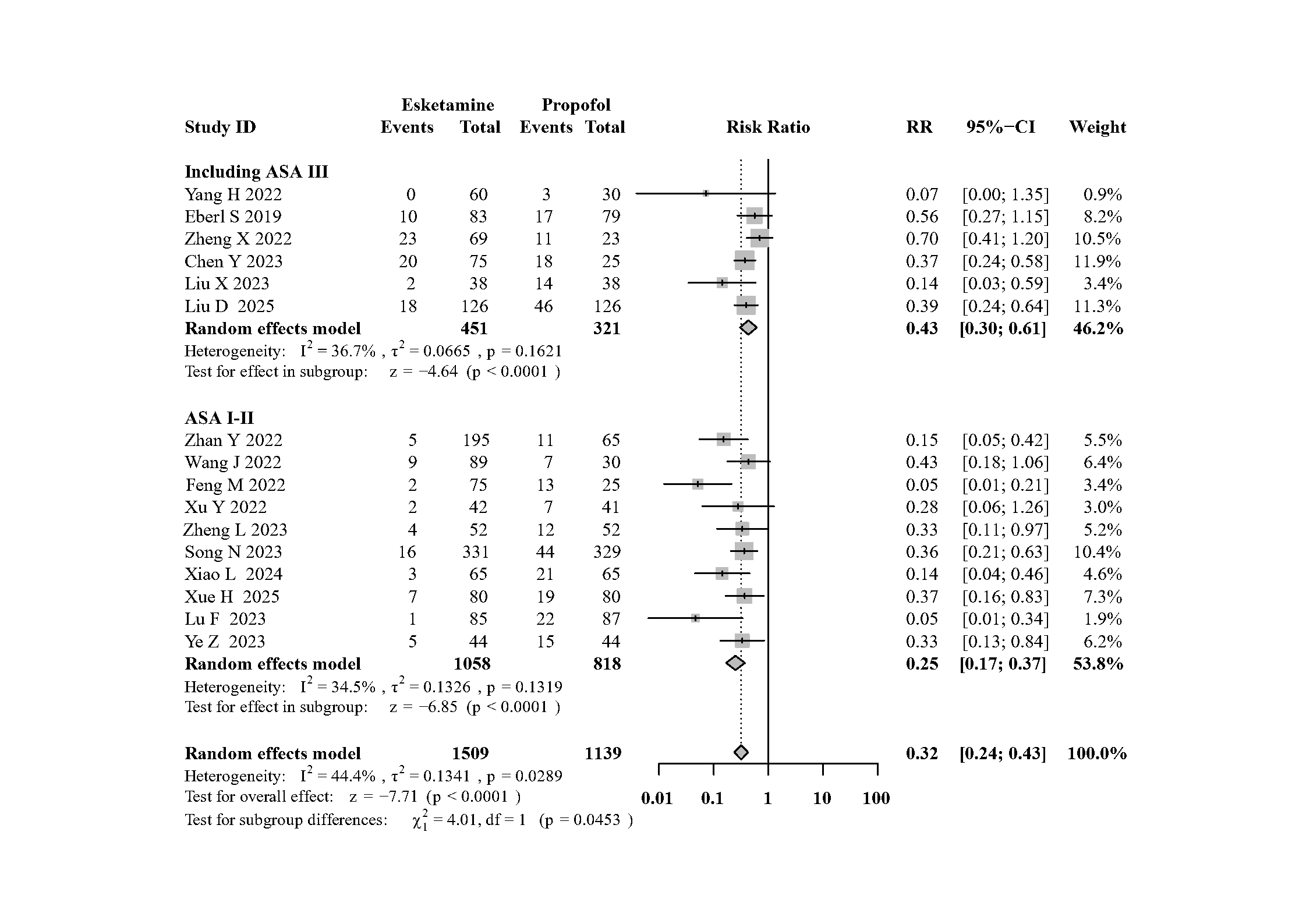


**Supplementary Figure 8. The subgroup analysis of the incidence of hypotension related to age.**


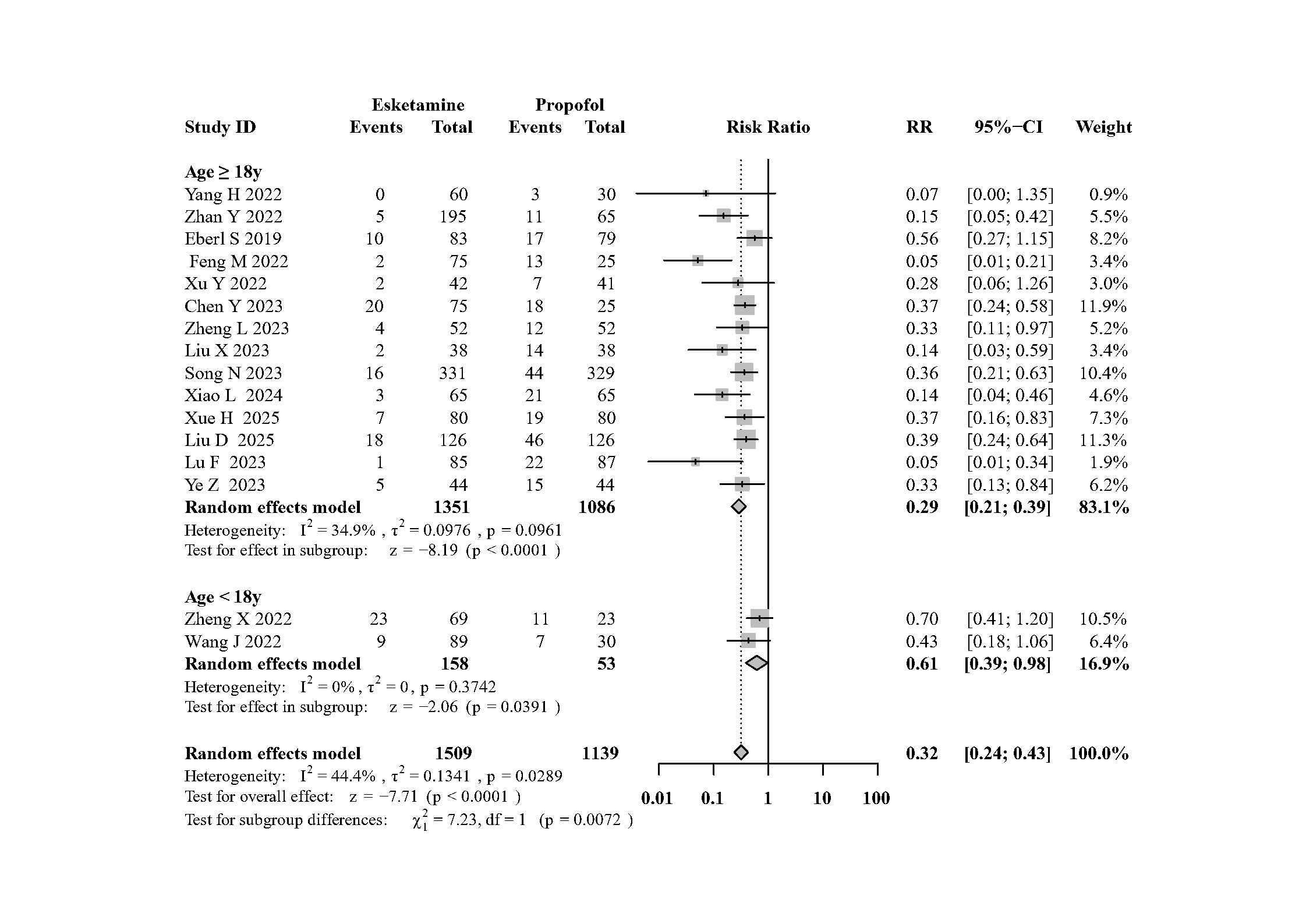


**Supplementary Figure 9. The subgroup analysis of the incidence of hypotension related to the dose of esketamine (≤0.2 mg/kg** **vs. >0.2 mg/kg).**

**
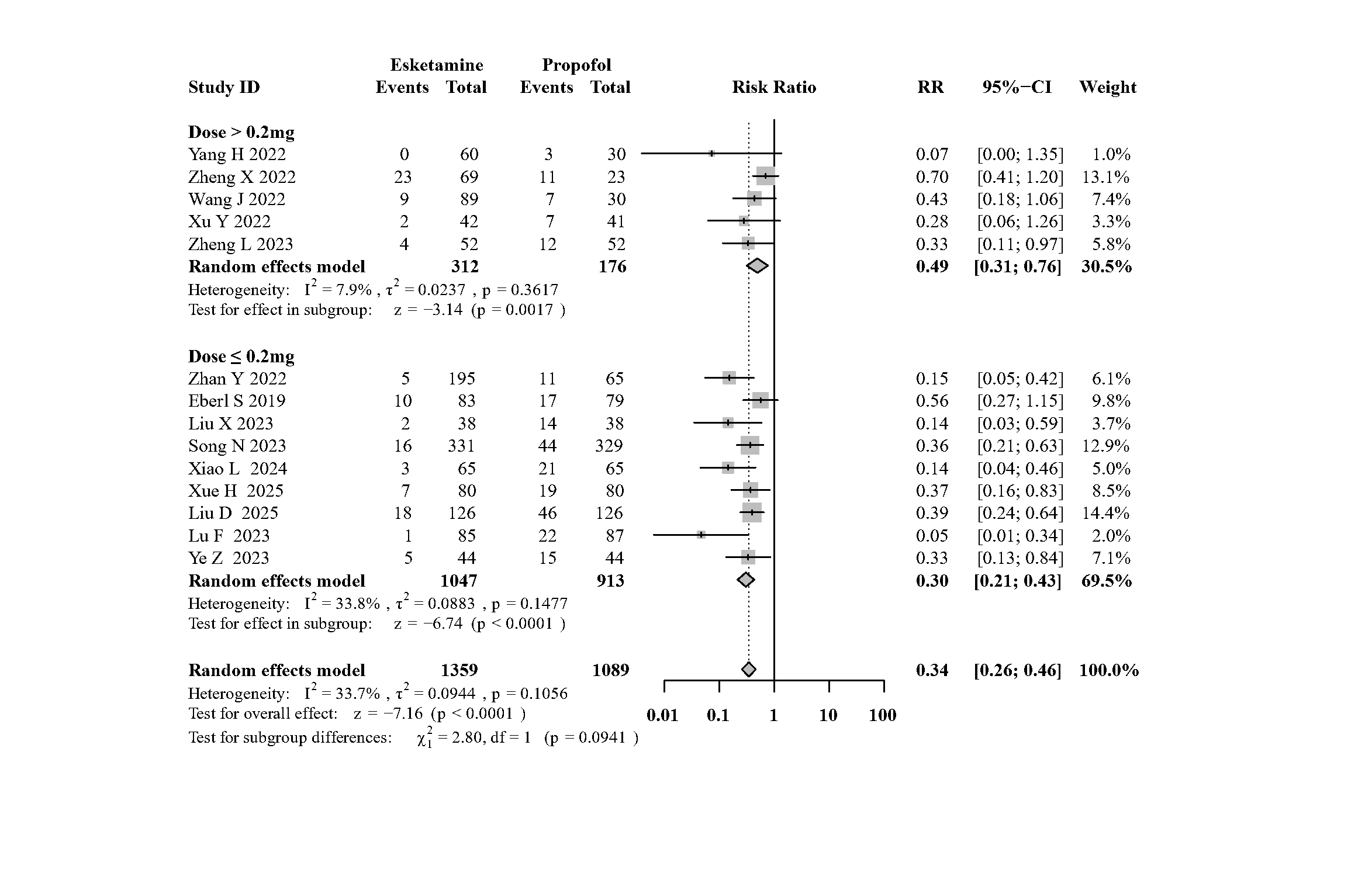
**

**Supplementary Figure 10. The subgroup analysis of the incidence of hypotension related to surgical location**

**
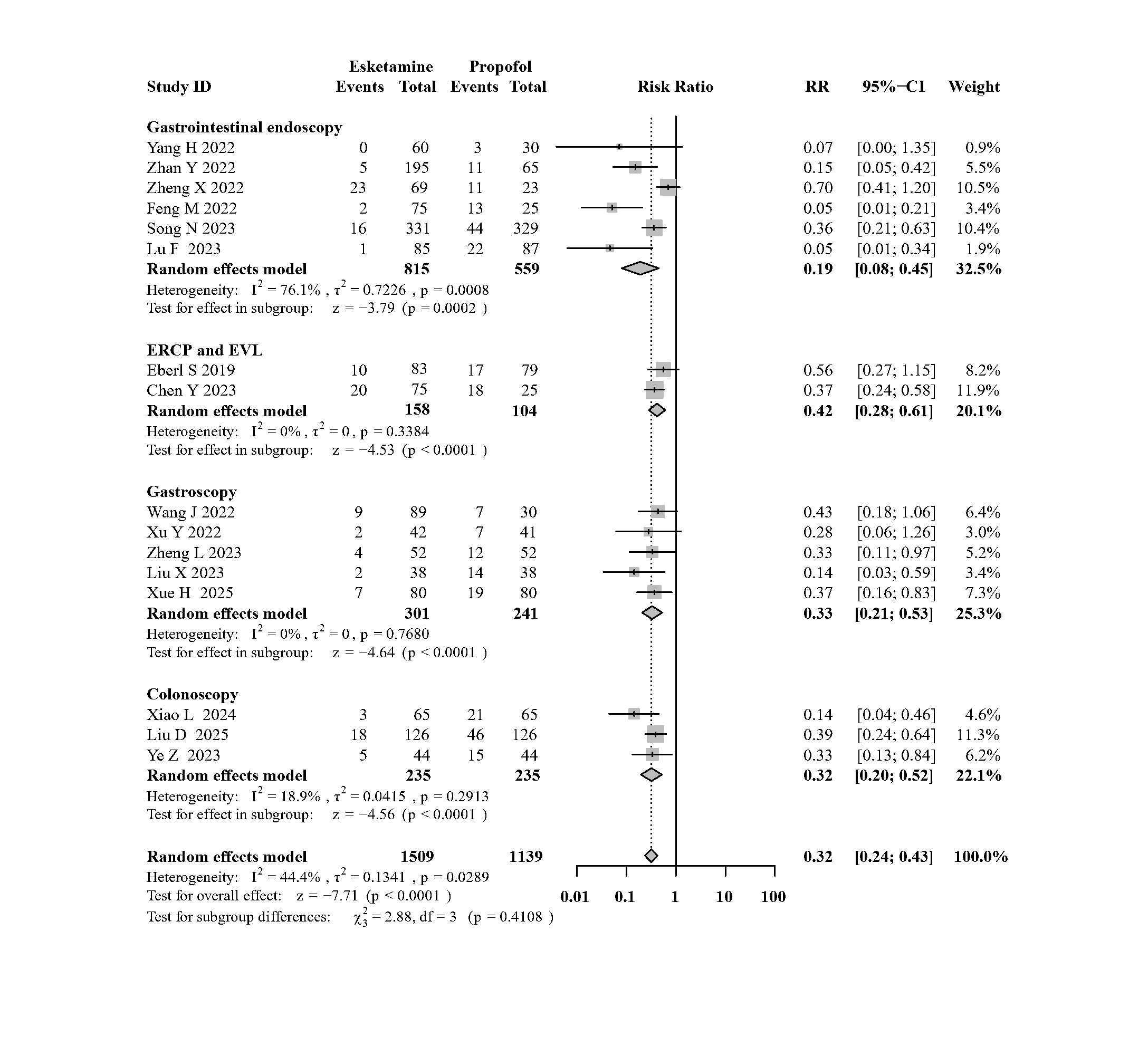
**

**Supplementary Figure 11. Forest plot of the propofol consumption (mg/kg).**


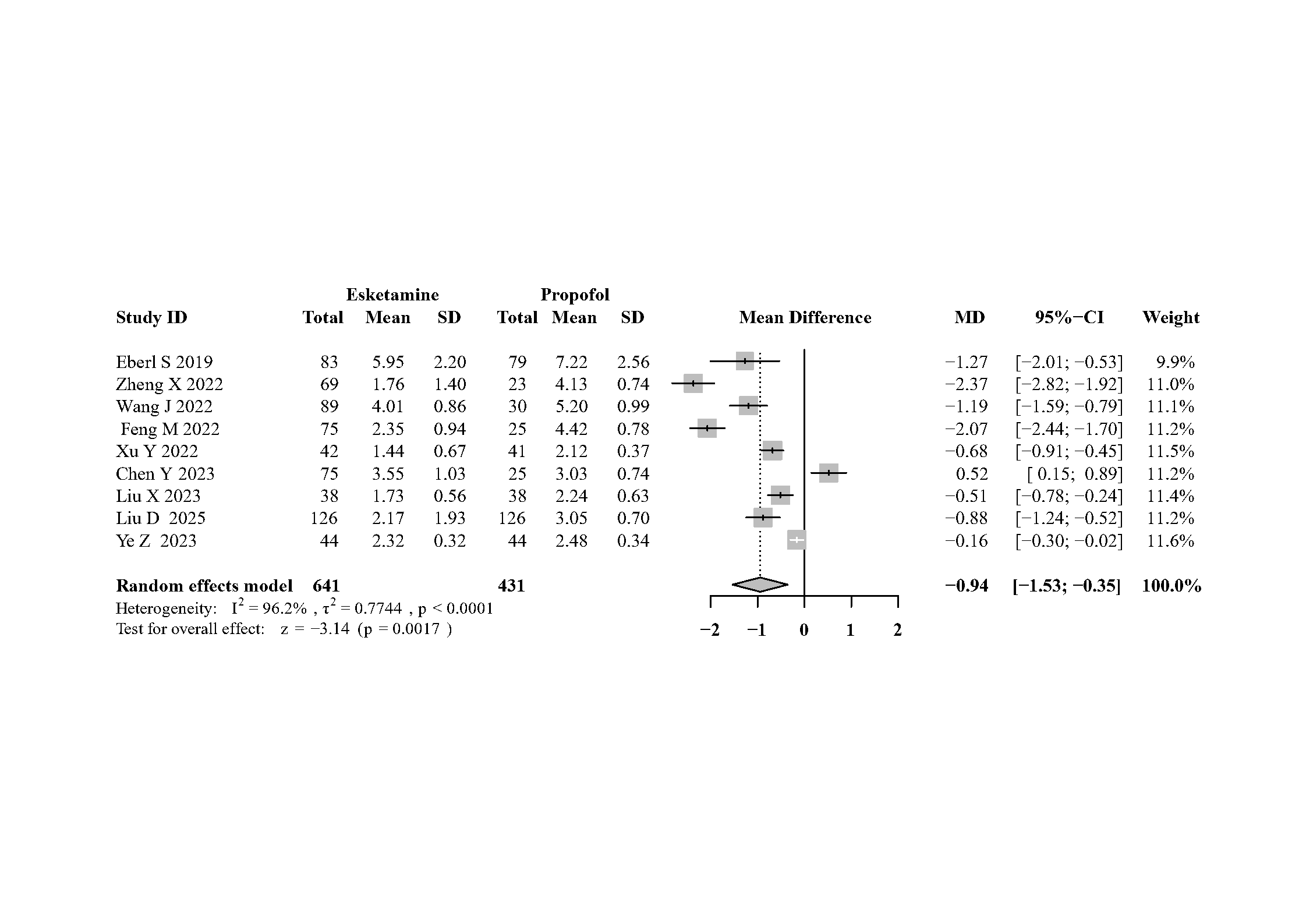


**Supplementary Figure 12. Forest plot of the incidence of hypertension.**


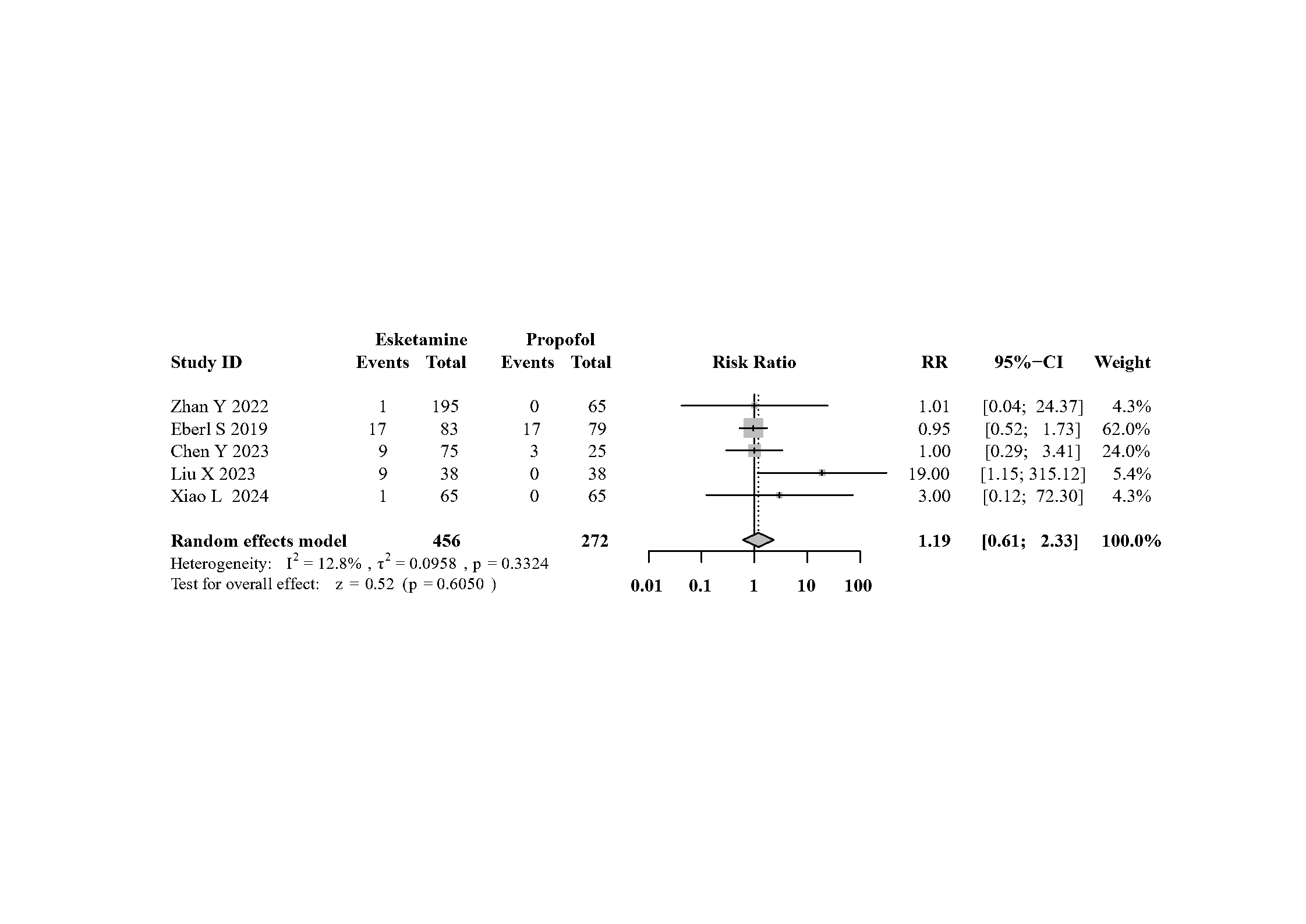


**Supplementary Figure 13. Forest plot of the incidence of arrhythmia.**


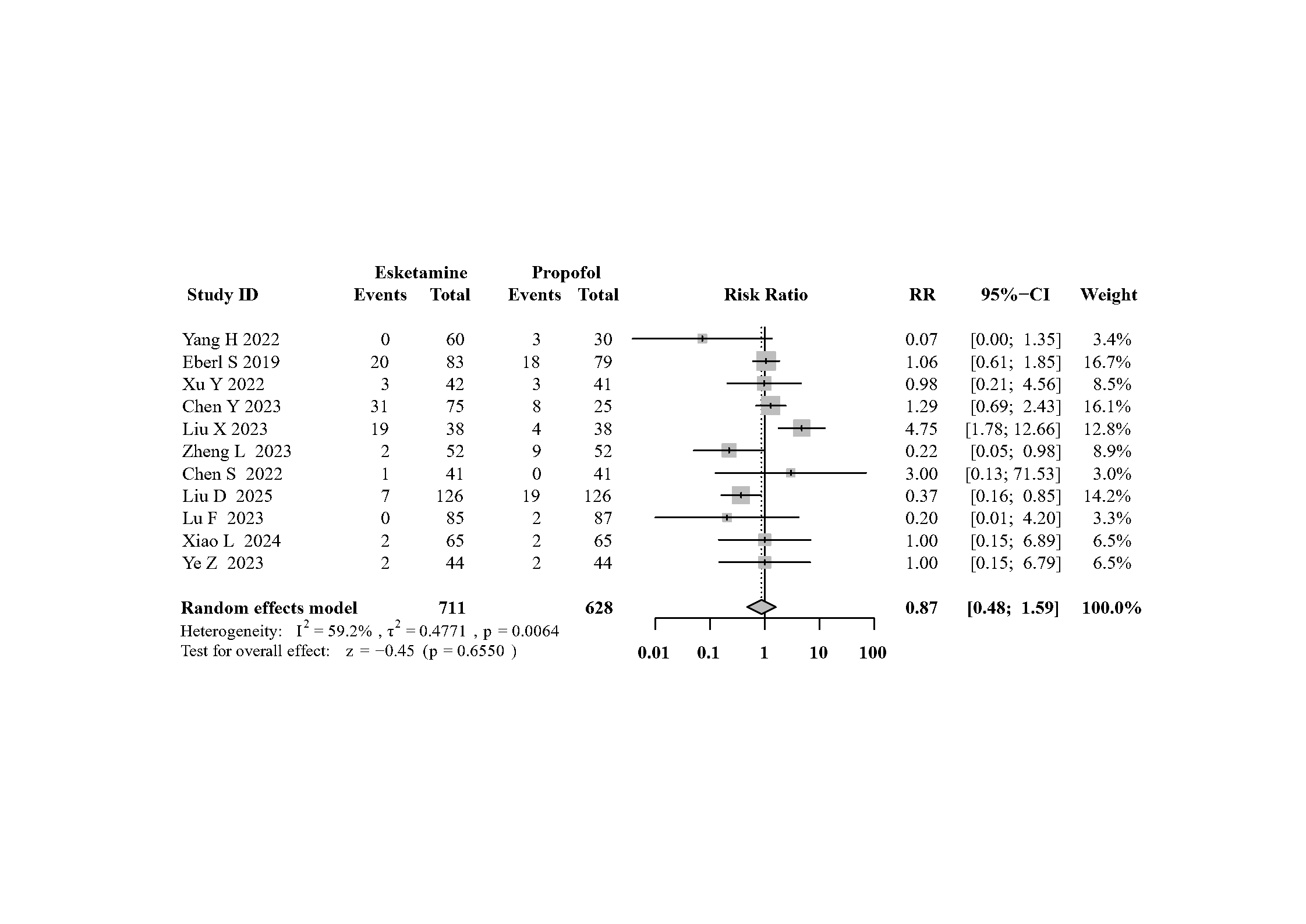


**Supplementary Figure 14. Forest plot of the incidence of PONV.**


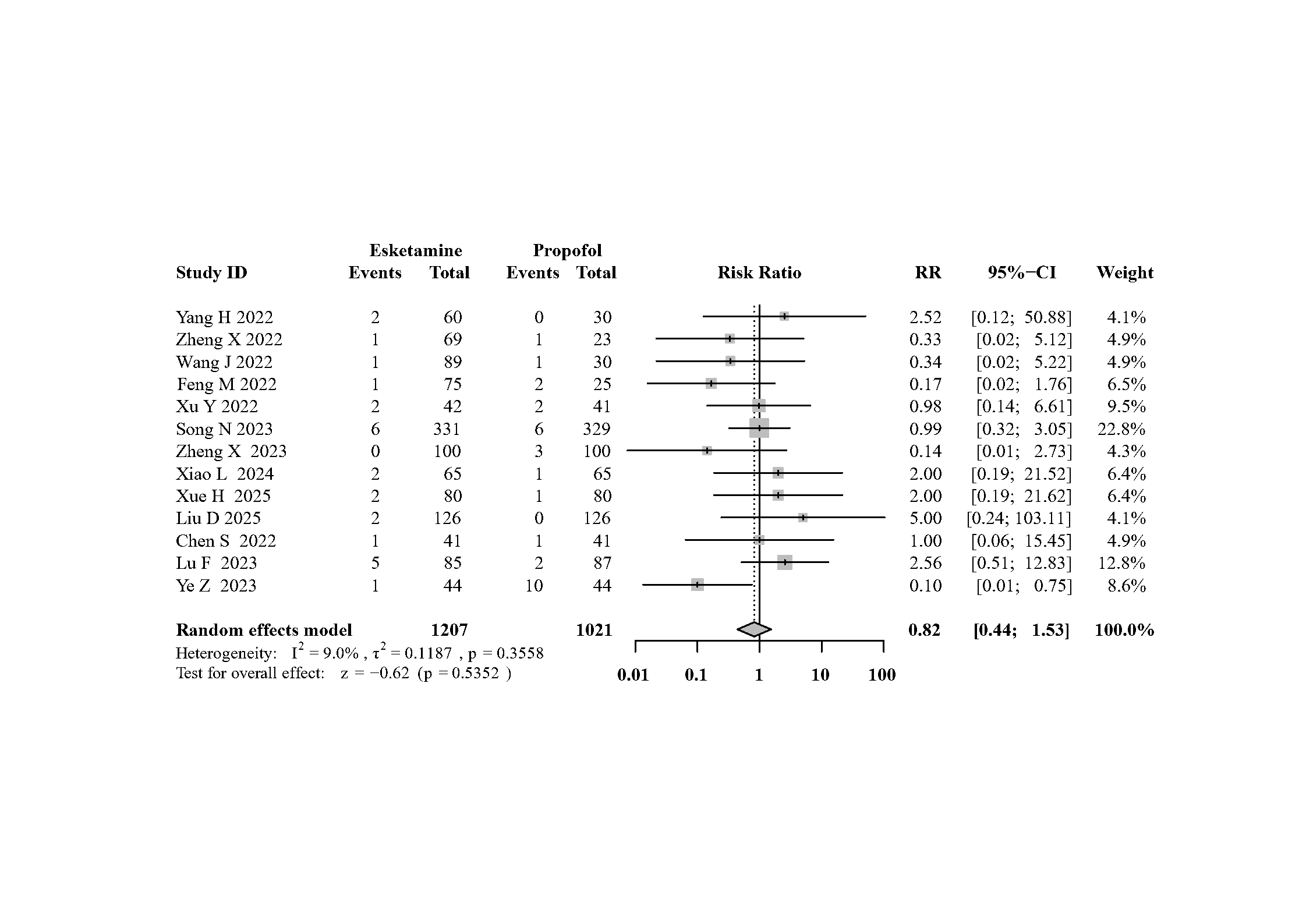


**Supplementary Figure 15. Forest plot of recovery time after surgery.**


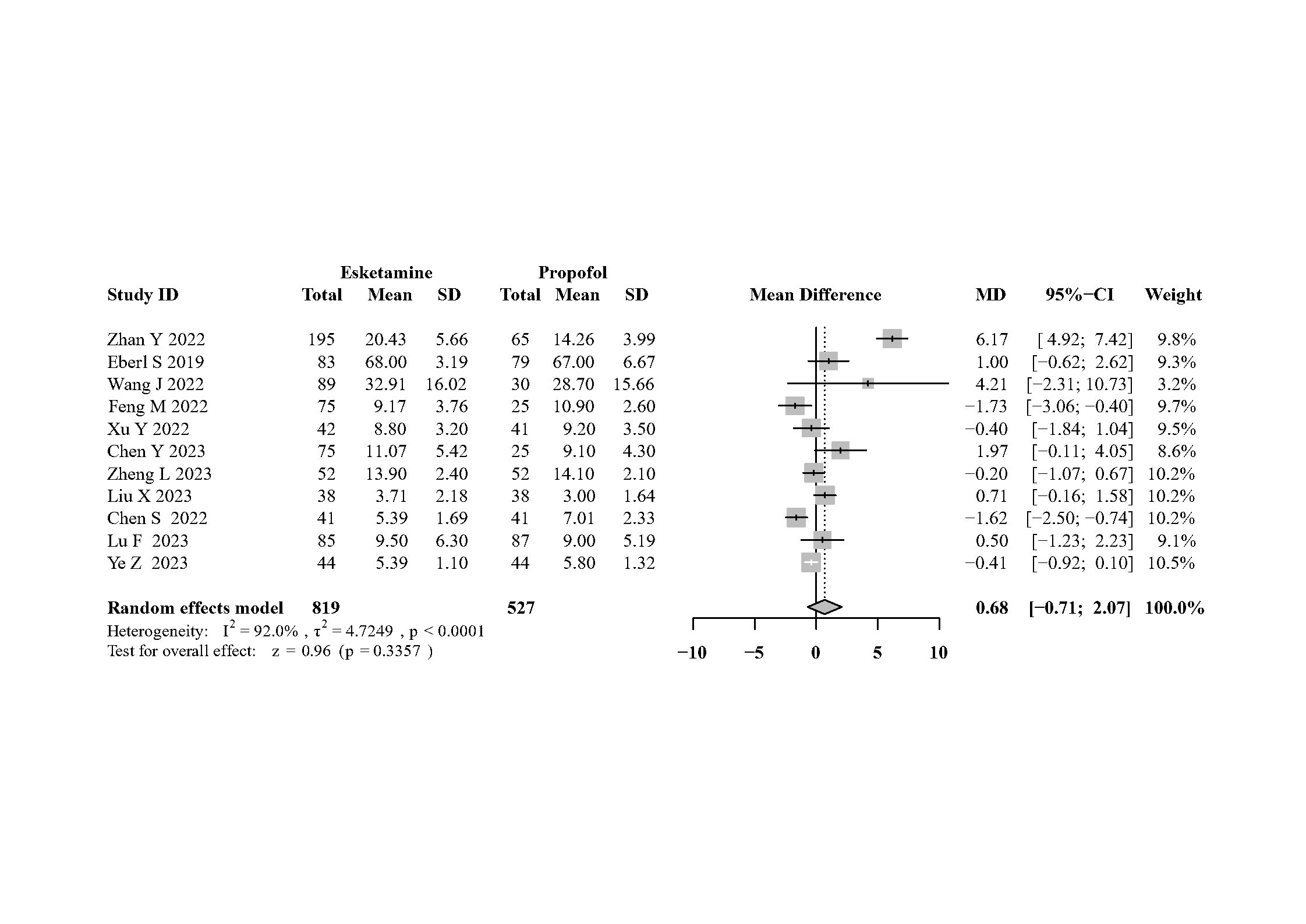


**Supplementary Figure 16. Forest plot of the incidence of dizziness after surgery.**


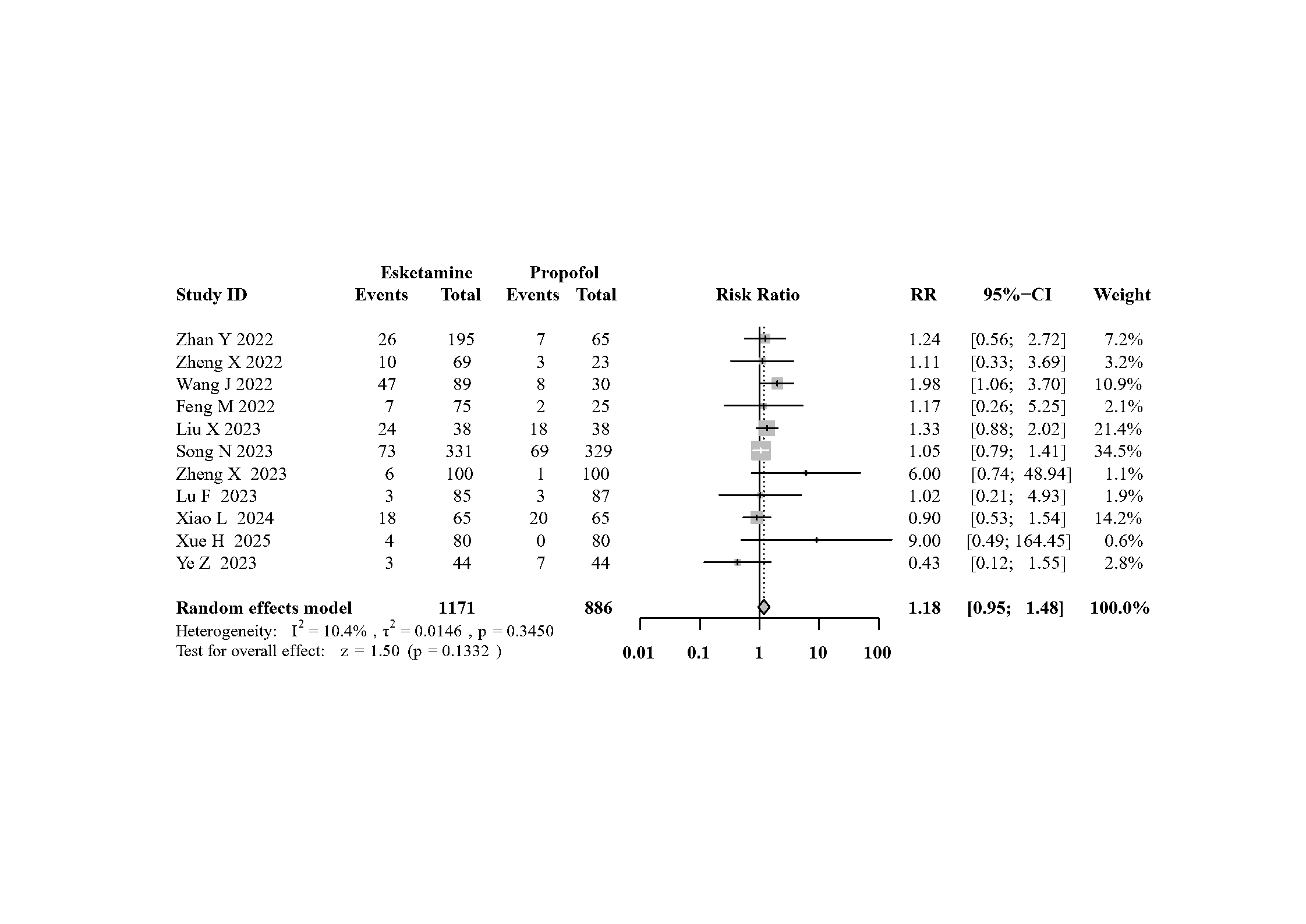


**Supplementary Figure 17. Bubble Patterns in Meta-Regression: Sample Size Impacts on Surgical Recovery Time.**


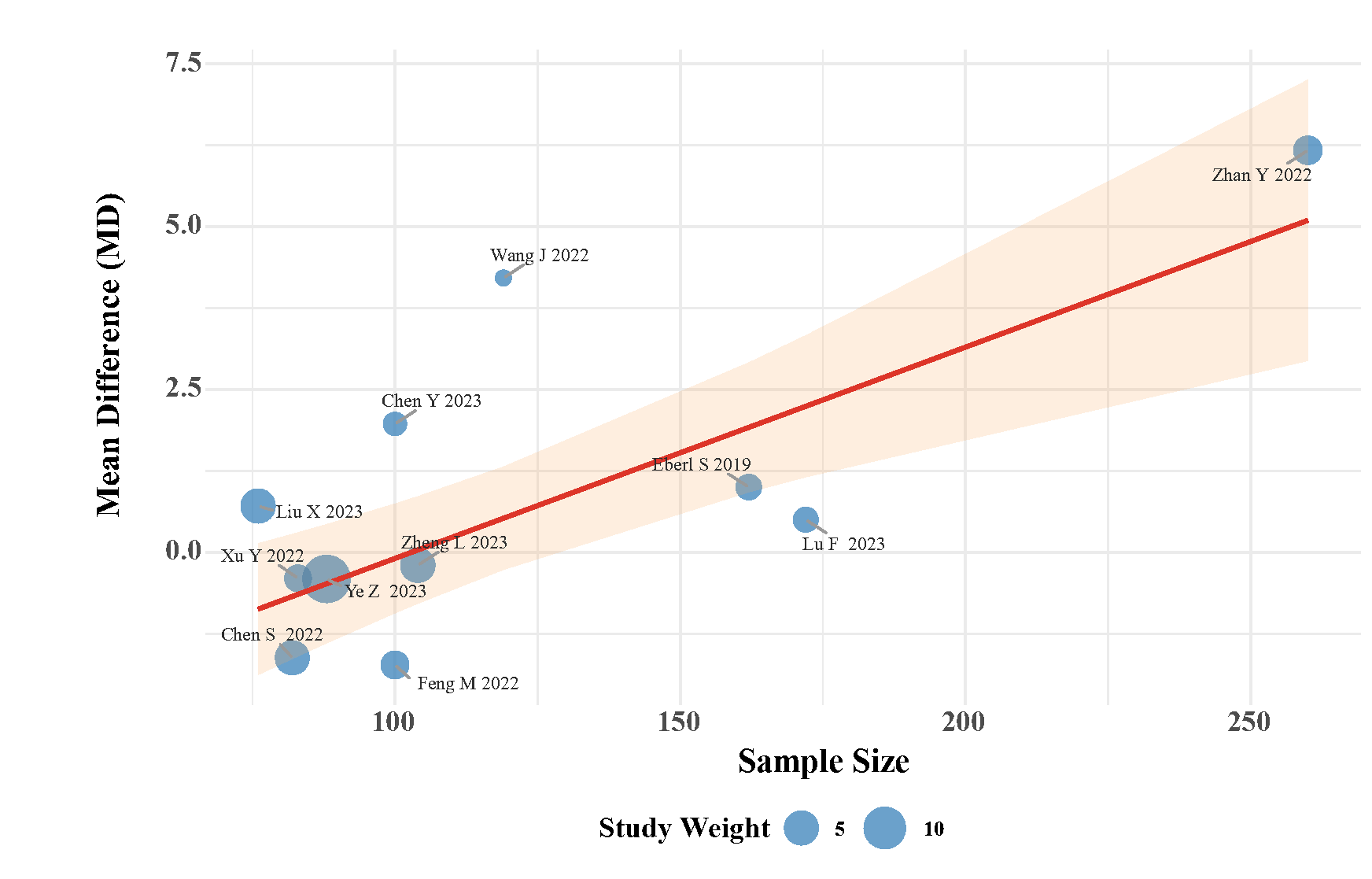


**Supplementary Figure 18. Forest plot of mean arterial pressure (MAP, mmHg) between the two groups after induction.**

**
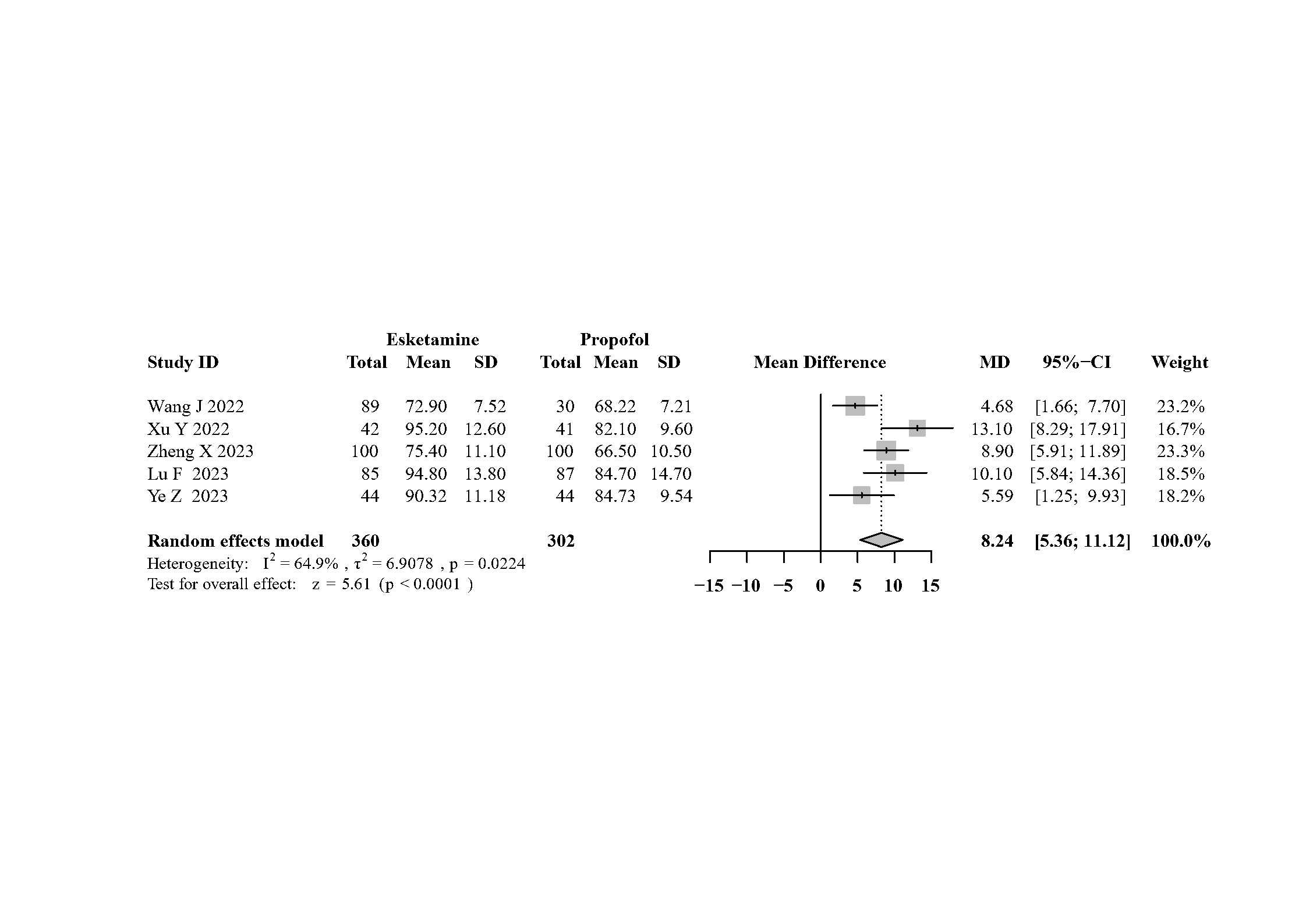
**

**Appendix. 1 Search strategies**

**Pubmed (****Aug 20, 2025)**

| **Number** | **Search concept** | **Results** |
| --- | --- | --- |
| 1 | esketamine | 1,729 |
| 2 | s-ketamine | 2,555 |
| 3 | (S)-2-(o-chlorophenyl)-2-(methylamino)cyclohexanone | 2 |
| 4 | "Esketamine" [Supplementary Concept] | 833 |
| 5 | sedation | 61,766 |
| 6 | sedative surgery | 29,764 |
| 7 | "Hypnotics and Sedatives"[Mesh] | 34,566 |
| 8 | painless | 21,755 |
| 9 | endoscopy | 500,912 |
| 10 | endoscopes | 599,700 |
| 11 | "Endoscopy"[Mesh] OR "Capsule Endoscopy"[Mesh] OR "Endoscopy, Digestive System"[Mesh] OR "Endoscopy, Gastrointestinal"[Mesh] OR "Endosonography"[Mesh] OR "Single-Balloon Enteroscopy"[Mesh] OR "Natural Orifice Endoscopic Surgery"[Mesh] | 443,805 |
| 12 | (((esketamine) OR (s-ketamine)) OR ((S)-2-(o-chlorophenyl)-2-(methylamino)cyclohexanone)) OR ("Esketamine" [Supplementary Concept]) | 2,556 |
| 13 | ((((((sedative surgery) OR (sedation)) OR ("Hypnotics and Sedatives"[Mesh])) OR (painless)) OR (endoscopy)) OR (endoscopes)) OR ("Endoscopy"[Mesh] OR "Capsule Endoscopy"[Mesh] OR "Endoscopy, Digestive System"[Mesh] OR "Endoscopy, Gastrointestinal"[Mesh] OR "Endosonography"[Mesh] OR "Single-Balloon Enteroscopy"[Mesh] OR "Natural Orifice Endoscopic Surgery"[Mesh]) | 719,228 |
| 14 | ((((esketamine) OR (s-ketamine)) OR ((S)-2-(o-chlorophenyl)-2-(methylamino)cyclohexanone)) OR ("Esketamine" [Supplementary Concept])) AND (((((((sedative surgery) OR (sedation)) OR ("Hypnotics and Sedatives"[Mesh])) OR (painless)) OR (endoscopy)) OR (endoscopes)) OR ("Endoscopy"[Mesh] OR "Capsule Endoscopy"[Mesh] OR "Endoscopy, Digestive System"[Mesh] OR "Endoscopy, Gastrointestinal"[Mesh] OR "Endosonography"[Mesh] OR "Single-Balloon Enteroscopy"[Mesh] OR "Natural Orifice Endoscopic Surgery"[Mesh])) | 390 |

**Web Of Science (Aug 20, 2025)**

| **Number** | **Search concept** | **Results** |
| --- | --- | --- |
| 1 | TS=(esketamine) and Preprint Citation Index (Exclude – Database) | 2,350 |
| 2 | TS=(s-ketamine) and Preprint Citation Index (Exclude – Database) | 1,506 |
| 3 | TS=((S)-2-(o-chlorophenyl)-2-(methylamino)cyclohexanone) and Preprint Citation Index (Exclude – Database) | 1 |
| 4 | #1 OR #2 OR #3 and Preprint Citation Index (Exclude – Database) | 3,574 |
| 5 | TS=(painless) and Preprint Citation Index (Exclude – Database) | 33,879 |
| 6 | TS=(sedation) and Preprint Citation Index (Exclude – Database) | 89,431 |
| 7 | TS=(sedative surgery) and Preprint Citation Index (Exclude – Database) | 9,877 |
| 8 | TS=(endoscopy) and Preprint Citation Index (Exclude – Database) | 255,419 |
| 9 | TS=(endoscopes) and Preprint Citation Index (Exclude – Database) | 150,345 |
| 10 | #5 OR #6 OR #7 OR #8 OR #9 and Preprint Citation Index (Exclude – Database) | 501,172 |
| 11 | #4 AND #10 and Preprint Citation Index (Exclude – Database) | 452 |

**Cochrane (Aug 20, 2025)**

| **Number** | **Search Concept** | **Results** |
| --- | --- | --- |
| 1 | esketamine | 1,745 |
| 2 | s-ketamine | 527 |
| 3 | #1 OR #2 | 2,204 |
| 4 | painless | 3,353 |
| 5 | sedation | 27,688 |
| 6 | sedative surgery | 2,338 |
| 7 | endoscope | 2,032 |
| 8 | endoscopy | 22,984 |
| 9 | MeSH descriptor: [Endoscopy] explode all trees | 26,622 |
| 10 | MeSH descriptor: [Endoscopes] explode all trees | 1,673 |
| 11 | MeSH descriptor: [Conscious Sedation] explode all trees | 1,712 |
| 12 | Hypnotics and Sedatives | 4,971 |
| 13 | MeSH descriptor: [Hypnotics and Sedatives] explode all trees | 4,895 |
| 14 | #4 OR #5 OR #6 OR #7 OR #8 OR #9 OR #10 OR #11 OR #12 OR #13 | 74,068 |
| 15 | #3 AND #14 | 600 |

**Chinese database (Aug 20, 2025)**

| **Name** | **Search Concept** | **Results** |
| --- | --- | --- |
| CNKI | ( (SU='艾司氯胺酮' OR SU='艾氯胺酮' OR SU='S-氯胺酮' OR TI='艾司氯胺酮' OR KY='艾司氯胺酮') )  AND  (SU='镇静' OR SU='麻醉' OR SU='无痛')  AND  ( (SU='胃肠镜' OR SU='消化内镜' OR SU='胃镜' OR SU='肠镜' OR SU='结肠镜' OR SU='内镜检查') OR TI='无痛胃肠镜' ) | 91 |
| VIP Database | ( (M=艾司氯胺酮 OR M=艾氯胺酮 OR M=S-氯胺酮 OR T=艾司氯胺酮 OR K=艾司氯胺酮) ) AND (M=镇静 OR M=麻醉 OR M=无痛) AND (M=胃肠镜 OR M=消化内镜 OR M=胃镜 OR M=肠镜 OR M=结肠镜 OR M=内镜检查) | 62 |
| Wanfang  Database | ( 主题:(艾司氯胺酮) or 主题:(艾氯胺酮) or 主题:(S-氯胺酮) ) and ( 主题:(镇静) or 主题:(麻醉) or 主题:(无痛) ) and ( 主题:(胃肠镜) or 主题:(消化内镜) or 主题:(胃镜) or 主题:(肠镜) or 主题:(结肠镜) or 主题:(内镜检查) ) | 168 |

**Reference**

1. Guyatt GH, Oxman AD, Kunz R, Brozek J, Alonso-Coello P, Rind D, Devereaux P, Montori VM, Freyschuss B, Vist G: **GRADE guidelines 6. Rating the quality of evidence—imprecision**. *Journal of clinical epidemiology* 2011, **64**(12):1283-1293.

2. Guyatt G, Oxman AD, Akl EA, Kunz R, Vist G, Brozek J, Norris S, Falck-Ytter Y, Glasziou P, DeBeer H *et al*: **GRADE guidelines: 1. Introduction-GRADE evidence profiles and summary of findings tables**. *J Clin Epidemiol* 2011, **64**(4):383-394.

3. Chen J, Zou X, Hu B, Yang Y, Wang F, Zhou Q, Shen M: **Effect of different doses of esketamine compared with fentanyl combined with propofol on hypotension in patients undergoing painless abortion surgery: a prospective, randomized, double-blind controlled clinical trial**. *Bmc Anesthesiology* 2022, **22**(1).

4. Zheng X-S, Shen Y, Yang Y-Y, He P, Wang Y-T, Tao Y-Y, Zheng J-J, Sun Y: **ED50 and ED95 of propofol combined with different doses of esketamine for children undergoing upper gastrointestinal endoscopy: A prospective dose-finding study using up-and-down sequential allocation method**. *J Clin Pharm Ther* 2022, **47**(7):1002-1009.

5. Zhan Y, Liang S, Yang Z, Luo Q, Li S, Li J, Liang Z, Li Y: **Efficacy and safety of subanesthetic doses of esketamine combined with propofol in painless gastrointestinal endoscopy: a prospective, double-blind, randomized controlled trial**. *Bmc Gastroenterology* 2022, **22**(1).

6. Zhong Y, Jiang M, Wang YS, Su TT, Lv YZ, Fan ZQ, Ning HY, Yang YL, Chen YH, Xie YB: **Evaluating efficacy and safety of sub-anesthetic dose esketamine as an adjuvant to propofol/remifentanil analgosedation and spontaneous respiration for children flexible fibreoptic bronchoscopy: a prospective, double-blinded, randomized, and placebo-controlled clinical trial**. *Front Pharmacol* 2023, **14**:12.

7. Guyatt GH, Oxman AD, Kunz R, Woodcock J, Brozek J, Helfand M, Alonso-Coello P, Glasziou P, Jaeschke R, Akl EA: **GRADE guidelines: 7. Rating the quality of evidence—inconsistency**. *Journal of clinical epidemiology* 2011, **64**(12):1294-1302.

8. Wang J, Hu W, Zhao X, Ren W, Huang X, Zhang B: **Sedative effect and safety of different doses of S-ketamine in combination with propofol during gastro-duodenoscopy in school-aged children: a prospective, randomized study**. *Bmc Anesthesiology* 2022, **22**(1).

9. Zheng XS, Shen Y, Yang YY, He P, Wang YT, Tao YY, Zheng JJ, Sun Y: **ED(50) and ED(95) of propofol combined with different doses of esketamine for children undergoing upper gastrointestinal endoscopy: A prospective dose-finding study using up-and-down sequential allocation method**. *J Clin Pharm Ther* 2022, **47**(7):1002-1009.

10. Liu X, Xiao Q, Zhuang S: **Comparison of propofol-esketamine versus propofol for anesthesia in gastroscopy: a double-blind, randomized controlled clinical trial**. *Frontiers in Medicine* 2023, **10**.

11. Eberl S, Koers L, van Hooft J, de Jong E, Hermanides J, Hollmann MW, Preckel B: **The effectiveness of a low-dose esketamine versus an alfentanil adjunct to propofol sedation during endoscopic retrograde cholangiopancreatography: A randomised controlled multicentre trial**. *Eur J Anaesthesiol* 2020, **37**(5):394-401.

12. Yang H, Zhao Q, Chen H-Y, Liu W, Ding T, Yang B, Song J-C: **The median effective concentration of propofol with different doses of esketamine during gastrointestinal endoscopy in elderly patients: A randomized controlled trial**. *British Journal of Clinical Pharmacology* 2022, **88**(3):1279-1287.
